# Toward collaborative open data science in metabolomics using Jupyter Notebooks and cloud computing

**DOI:** 10.1007/s11306-019-1588-0

**Published:** 2019-09-14

**Authors:** Kevin M. Mendez, Leighton Pritchard, Stacey N. Reinke, David I. Broadhurst

**Affiliations:** 1 Centre for Metabolomics & Computational Biology, School of Science, Edith Cowan University, Joondalup, 6027 Australia; 2 Strathclyde Institute of Pharmacy & Biomedical Sciences, University of Strathclyde, Cathedral Street, Glasgow, G1 1XQ Scotland, UK

**Keywords:** Open access, Reproducibility, Data science, Statistics, Cloud computing, Jupyter

## Abstract

**Background:**

A lack of transparency and reporting standards in the scientific community has led to increasing and widespread concerns relating to reproduction and integrity of results. As an omics science, which generates vast amounts of data and relies heavily on data science for deriving biological meaning, metabolomics is highly vulnerable to irreproducibility. The metabolomics community has made substantial efforts to align with FAIR data standards by promoting open data formats, data repositories, online spectral libraries, and metabolite databases. Open data analysis platforms also exist; however, they tend to be inflexible and rely on the user to adequately report their methods and results. To enable FAIR data science in metabolomics, methods and results need to be transparently disseminated in a manner that is rapid, reusable, and fully integrated with the published work. To ensure broad use within the community such a framework also needs to be inclusive and intuitive for both computational novices and experts alike.

**Aim of Review:**

To encourage metabolomics researchers from all backgrounds to take control of their own data science, mould it to their personal requirements, and enthusiastically share resources through open science.

**Key Scientific Concepts of Review:**

This tutorial introduces the concept of interactive web-based computational laboratory notebooks. The reader is guided through a set of experiential tutorials specifically targeted at metabolomics researchers, based around the Jupyter Notebook web application, GitHub data repository, and Binder cloud computing platform.

**Electronic supplementary material:**

The online version of this article (10.1007/s11306-019-1588-0) contains supplementary material, which is available to authorized users.

## Introduction

Historically, journal articles have been the primary medium for sharing new scientific research. The intent of article content, and the corresponding review process, is to ensure adequate evidence of reproducibility; however, a recent report highlights increasing and widespread concerns relating to reproduction and integrity of results, with 52% of responding scientists agreeing there is a significant ‘crisis’ of reproducibility (Baker 2016). We and many others in the metabolomics community hold the view that a lack of transparency and incomplete reporting has led to significant misinterpretation of data and a lack of trust in reported results (Broadhurst and Kell 2006; Considine et al. 2017; Goodacre et al. 2007; Spicer et al. 2017; Xia et al. 2013). A mechanism that may address these concerns is for the scientific community to take advantage of new online publishing media and associated data services, encouraging open science that recognises and aligns with the FAIR (Findable, Accessible, Interoperable, and Reusable) data principles (Wilkinson et al. 2016).

This concern in metabolomics and other post-genomic platforms is a consequence of their success. The unprecedented rate at which new mathematical algorithms and computational tools are developed and adopted means that published findings are increasingly the sole result of computationally intensive data processing (Teschendorff 2019). Advances in measurement technologies continue to generate ever increasing volumes of high-throughput data, which in turn require multidisciplinary expertise as ever more complex and elaborate statistical methods are used to infer generalisable biological associations (Pinu et al. 2019) and sophisticated visualization tools are used to make large datasets more understandable (Gehlenborg et al. 2010; Holten 2006). Indeed, recent advances in machine learning algorithms combined with improved visualisation strategies now allow researchers to integrate and interrogate multi-omic data sets of a size that was unmanageable 5 years ago (for example, Lee et al. 2019; Reinke et al. 2018; Rohart et al. 2017). For science to continue to move forward under this deluge of data while avoiding technical debt and maintaining reproducibility, the processes surrounding research data management, storage, analysis, and presentation need to be agile, transparent, reusable, and recoverably attached to published work. A further challenge is that, to gain broad adoption and be used by busy practising researchers, frameworks conforming to these requirements must also be intuitive and accessible to users who may have limited computational expertise.

Over the last several years, the metabolomics community has made bold strides towards adopting FAIR standards for data including: development of vendor-independent raw data formats (such as mzXML) (Pedrioli et al. 2004); open access data repositories such as MetaboLights (Haug et al. 2012), and Metabolomics Workbench (Sud et al. 2016); open access online spectral reference libraries such as METLIN (Smith et al. 2005), mzCloud (https://www.mzcloud.org/), and MassBank (Horai et al. 2010); and online databases for metabolite identification and biochemical association such as HMDB (Wishart et al. 2018). These resources and others like them are fundamental to the future integrity of metabolomics as a science. It is well-recognised that open, interoperable datasets are essential for progress, and the computational tools and methods that convert, step-by-step, metabolite data to biochemical meaning also need to be FAIR (Wilkinson et al. 2016).

Numerous groups within the metabolomics community actively work to standardise computational workflows and provide online tools for statistical analysis (some recent advances are discussed later in this paper). However, a common characteristic of many computational frameworks encountered by researchers is a tendency to be prescriptive, in that they provide a restricted set of well-curated “plug and play” computational stepping stones that enable only limited choices within the workflow framework. These constraints limit the ability of a user to fully exploit the provided methodologies, or to explore and develop new analytical approaches. Presentation of analysis steps as pluggable “black box” approaches is convenient but diminishes opportunities for education and understanding of the analysis methods being used. To fully embrace the concept of ‘open data science’ the metabolomics community needs an open and easily accessible computational environment for rapid collaboration and experimentation.

**Fig. 1:**
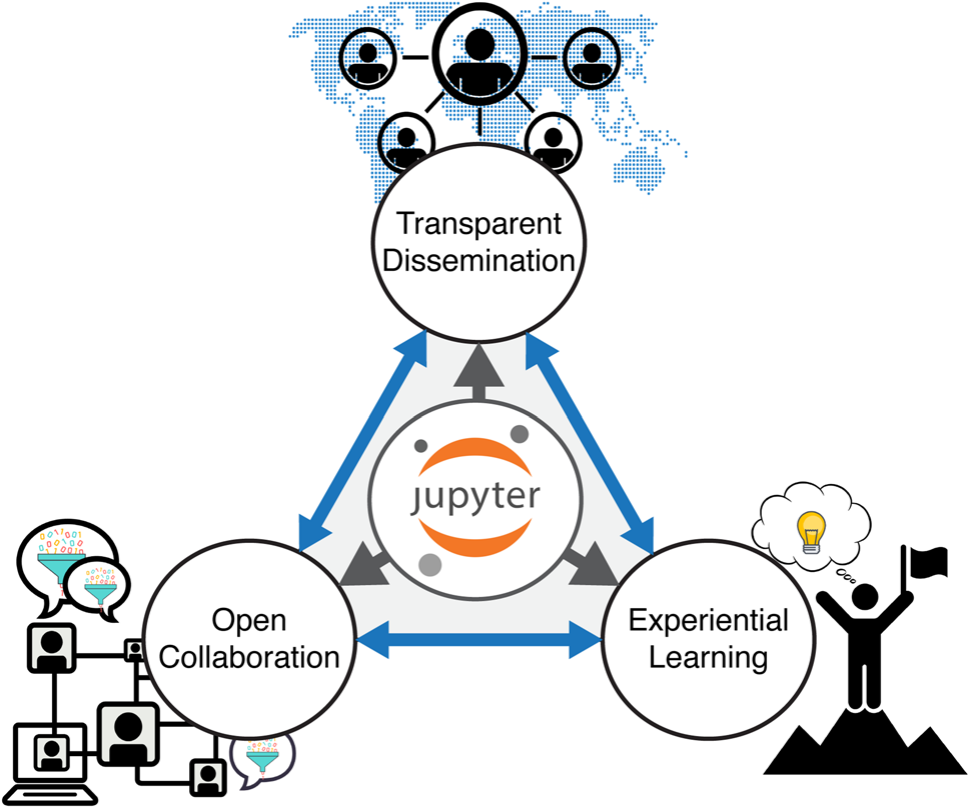
Applications for Jupyter Notebooks in the postgenomic community. Open virtual notebooks have three main, non-mutually exclusive, applications. First, they provide an efficient means for transparent dissemination of methods and results, thereby enabling alignment with FAIR data principles. Second, they provide a central and interactive platform that facilitates open collaboration to develop methodology and perform data analysis. Finally, their interactive and easily deployable framework can drive experiential learning opportunities for computational novices to develop their own skills and better understand metabolomics data analysis

The subject of this tutorial review is a practical open-science solution to this problem that balances ease-of-use and flexibility, specifically targeted to novice metabolomic data scientists. This solution takes the form of ‘computational lab books’, such as Jupyter Notebooks (Kluyver et al. 2016), that have a diverse range of overlapping potential applications in the post-genomic research community (Fig. 1). Firstly, they enable open collaboration by providing a central platform for researchers to cooperatively develop methodology and perform data analysis. Secondly, they provide a means for transparent dissemination of a finished study or product. In a formal context computational lab books can comprise supplemental material extending the reach of a publication that enables readers to rapidly recreate data analyses and figures for themselves. In an informal context, they can provide a polished “showcase” that allows users to interact with and understand the functionality of underlying algorithms. Finally, the inherent promotion of direct user interaction enables experiential learning opportunities, where the user develops their understanding and skills through active experimentation, reflective observation, and abstract conceptualisation (Kolb 1984).

In this review, we provide a brief overview of current data science frameworks relevant to the metabolomics community, corresponding barriers to achieving open science, and finally a practical solution in the form of the computational lab notebook, where code, prose and figures are combined into an interactive notebook that can be published online and accessed in a modern web browser through cloud computing. We present a set of experiential learning tutorials introducing the Jupyter Notebook framework, specifically tailored to the needs of a metabolomics researcher. The tutorials are designed in a hierarchy of complexity following Bloom’s taxonomy of educational learning objectives (Anderson et al. 2001). Tutorial one introduces the basic concepts of Jupyter Notebooks. Tutorial two encourages interactive learning using an existing metabolomics data science Jupyter notebook. Tutorial three establishes the framework in which the user can create a Jupyter notebook on a local computer. Tutorial four teaches the user how to create a simple notebook for their own data. Tutorial five explains how to publish and share a new Jupyter notebook in the cloud. The overarching aim of this document is to encourage metabolomics researchers from all backgrounds, possibly with little or no computational expertise, to seize the opportunity to take control of their own data science, mould it to their personal requirements, and enthusiastically share resources through open science.

## Background

A glossary of terms has been provided in Table 1 to help clarify technical terms used in this tutorial.

**Table 1:** Glossary of terms

Paper section	Term	Definition
1	Data repository	A platform (such as Metabolights or Metabolomics Workbench) used to store metadata and experimental data
2.1	Command line interface (CLI)	A user interface that is used to execute operating system functions using text
2.1	Graphical user interface (GUI)	A user interface that is used to execute operating system functions using graphical icons or other visual indicators
2.1	Integrated development environment (IDE)	A software application that provides an interface to write and test code (such as RStudio, PyCharm and Visual Studio Code). It typically includes basic tools such as a code editor, compiler, and a debugger
2.1	Containers	Self-contained units of software that package code, dependencies, system tools and system libraries. The purpose is to be reliably transferred between, and deployed on, various operating systems and infrastructures
2.1	JavaScript object notation (JSON) format	A lightweight data-interchange format commonly used for communication between a browser and server. Internally, Jupyter Notebooks are JSON files with the.ipynb extension
2.1	Packages	Units of shareable code that can be imported and used to provide additional functionality (such as matplotlib and scikit-learn)
2.1	Application programming interface (API)	A set of defined functions and protocols for interacting with the software or package
2.1	Kernel	The “computational engine” that runs and introspects the code contained in a notebook document. Jupyter supports a kernel for Python, as well as kernels for many other languages (such as R, Julia, Kotlin, etc.)
2.2	Version control	A documented history of changes made to a file, enabling step-by-step reproduction and reconstruction of its development
2.2	Code repository	A hosted archive (such as those at GitHub and BitBucket) of source code and supporting files.
3	Virtual environment	An isolated environment that contains a specific version of Python and dependencies
3.1.1	Distribution (Software)	A collection of software bundled together
3.1.1	Markdown	A lightweight markup language used to add and format plain text. It is used in Jupyter Notebooks within “Markdown” cells
3.1.3	Configuration file	A file used to set the initial settings and parameters for computer applications. It is used in Binder to build the virtual environment with specific dependencies
3.2.1	Text cell (Markdown cell)	A cell in the Jupyter Notebook used to write text (using the Markdown language)
3.2.1	Code cell	A cell in the Jupyter Notebook used to run code (such as Python code)
3.2.3	Sandbox (Software development)	A software environment typically used to run or test experimental code in isolation from the rest of the system
3.2.5	Dependencies	The packages (and versions) that are required to be installed to use the software. For Python, these are the packages that need to be imported at the start of the file
3.2.5	Channels (Specific to Anaconda)	The location where packages that are installed using conda are stored (such as conda-forge and bioconda)
3.2.5	README	A file (commonly markdown or text) used to communicate information to visitors about the repository (such as purpose, usage, and contributors)
3.2.5	Root directory	The directory (or folder) that is the highest level in a hierarchy

### Software tools and barriers to open science

Many statistical and data science software tools are available for use in metabolomics data analysis and visualisation. They can be classified as commercial (requiring a paid licence) or “free” (as in zero-cost) and, in either case, may be open-source (the underlying computer code is available for inspection) or proprietary (closed-source, code unavailable for inspection). The primary mode of interaction with the user may be via scripting, a command line (CLI), or a graphical (GUI) user interface.

Commercial, proprietary (closed-source) GUI software packages include Microsoft Excel, Minitab, SPSS, Unscrambler, and SIMCA (Umetrics). Tools like these generally offer the benefits of being user-friendly, stable and reliable platforms with well-documented resources, and have a high level of technical customer support. However, proprietary software can also lack methodological transparency because the source code is not freely available. When source code cannot be inspected a researcher’s ability to interrogate underlying algorithms, demonstrate correctness, diagnose problems, or improve the tool is limited. If the package prescribes an analytical workflow it may be difficult, or even impossible, to embed alternative third-party computational steps. If additional functionality is required users are dependent on the software’s developers to implement this, which may impose an additional expensive commercial transaction even in cases where the request is approved. It is also difficult to produce usable graphical interfaces that are also customisable by the user, so this kind of interface can be relatively inflexible and so constrain the researcher to a specific mode of working.

Command-line or script-based proprietary software packages such as MATLAB, SAS, and Stata overcome some of the limitations imposed by graphical interfaces and closed-source code by allowing third party code to be embedded, and implementation of alternative algorithms and arbitrary workflows by the researcher. In the case of MATLAB the source code of some or all of the proprietary tools is readable, which improves transparency of methods, and it is possible for the programmer to develop open custom graphical interfaces. However, even then open-source commercial packages can carry a significant financial cost limiting the ability of researchers, especially those in developing nations or on smaller budgets, to replicate results, adapt methods, or collaborate to develop better workflows. We consider that open-source “free” tools and applications will form the future basis of shareable research, as they enable the greatest possible degree of transparency and reproducibility.

Open-source GUI workflows providing simplified or user-friendly access to underlying programs and analytical tools have been developed to improve usability for scientists who have not yet acquired the programming skills necessary to write their own pipelines and workflows. Within the metabolomics community popular applications include: MetaboAnalyst (Xia and Wishart 2011), Galaxy-M (Davidson et al. 2016), and Workflow4Metabolomics (Giacomoni et al. 2015). Galaxy workflows provide a unified data visualisation and analysis environment that allows seamless (to the user) integration of multiple open-source software packages, and tools written in multiple programming languages (Afgan et al. 2018). These tools allow rapid construction, implementation, and sharing of standardised workflows, including integration with remote and local databases, without the need for programming skills. This provides a mechanism to ensure methodological consistency and precise reporting standards. Resources such as Galaxy simplify the user experience and enable flexible use of a wide range of open source tools.

Despite the many strengths of open-source GUI workflows such as Galaxy, they do not always provide users with a free choice of available data analysis methods. For example, unless the user has administrative rights on the server, the browser interface of Galaxy does not permit direct access to software package management. This restricts extension, modification, and development of workflows by the user. Although an arbitrary set of tools can in principle be “wrapped” by a researcher for use with Galaxy, there may be in practice only limited support for requests to implement a tool, especially when working on public servers. It is possible to implement arbitrary tools and processes in a locally-managed Galaxy instance with administrative control of the workflow service, but this requires investment of time, technical expertise, and local computational capacity, as well as carrying implications for long-term systems support and maintenance.

Even with a free choice of tools and algorithms, workflows implemented in GUI-based tools like Galaxy are “linear” in the sense that the browser interface imposes a process in which data passes through a sequential chain of operations. These interfaces are not well-suited to representing complex workflow logic, such as branches and loops that explore alternative approaches or parameter choices as part of the same analysis. This can inadvertently encourage a “black box” one-size-fits-all approach to analysis that may be of concern when the dataset is non-standard or, for example, when a statistical analysis requires customisation due to assumptions made by the model regarding the distribution of the input data. Incurious application of standardised GUI workflows with limited opportunity for experimentation can lead to inappropriate analytical strategies and unintentional misreporting of results. The linearity constraint is recognised by the Galaxy developers, who provide a programmatic Application Programming Interface (API) enabling automation of complex workflow logic, but this requires programming ability to use.

Another limitation of GUI workflow-based applications can be a lack of contextual annotation. With most interfaces the user must document separately why computational methods and parameter settings were chosen in a specific workflow. It is not typically possible through GUI workflow interfaces to embed the experimental context, explanation of methods, code, and figures into a single *live* (interactive) document. The formal reporting may then be reduced to a terse listing of steps and parameter values for generating data, tables and figures, rather than a more readable “literate programming” account of the analysis. This retrospective approach is sufficient and appropriate for standardised, repeated workflows that vary little from experiment to experiment, such as a mass spectrometry deconvolution workflow that converts a set of raw instrument files into an annotated table (e.g. XCMS → CAMERA → MetFrag). However, when a metabolomics scientist moves on to statistical analysis, multivariate machine learning, and data visualisation to extract and present a biologically-informative interpretation of the data, it is desirable to have an integrated, flexible data analysis environment that includes detailed annotation of analysis choices.

The most flexible data science solution is to conduct analyses in one or more high-level open-source programming languages such as C, Fortran, Java, Julia, Perl, Python, Octave, R, or Scala, that also support sophisticated statistical tools. Python and R have become especially popular languages in data science due to the availability of comprehensive, robust, and well-documented code libraries (modules/packages). Many statistical and machine learning packages are available for these languages (including bindings to Galaxy, which overcomes some of the GUI-based limitations of that platform), with strong data science community support (Lantz 2013; Müller and Guido 2017). However, these general-purpose languages may present novice (or non) data scientists with a forbiddingly steep learning curve, especially in comparison with GUI tools. To be most effective in these languages a researcher requires a basic understanding of computer programming to use the available code libraries in their specific field. There is an initial learning curve, but knowledge of a programming language is more generally useful and broadly applicable than familiarity with a specific software tool’s interface and can impact positively on many areas of research. Programming is increasingly recognised as a foundational skill for research and promoted at all levels from primary to postgraduate education (Passey 2017). The broad impact of this skillset throughout academic research, including arts and humanities, is recognised in the growing influence of training foundations such as The Carpentries (https://carpentries.org/) that aim to “[teach] researchers the computing skills they need to get more done in less time and with less pain.”

Several freely-available software tools bridge the gap between GUI interfaces and high-level languages by providing a user interface for researchers to develop their own code. For Python and R, integrated development environments (IDEs) such as PyCharm (Python), RStudio (R), and more general multi-language IDEs (e.g. Visual Studio Code, Komodo and Eclipse), provide additional tools for automating, testing and visualizing the process of writing scripts, programs and analysis workflows. These IDEs can simplify the learning and programming experience but are primarily designed for larger program and application development, rather than composing and sharing data analysis workflows. However, IDEs in general are extremely useful even to the novice programmer, and some prominent examples are specifically targeted towards data analysis, such as RStudio and JupyterLab.

Recently, several independent strands of general-purpose data science software development have been woven into practical solutions to the various limitations of the above frameworks. Firstly, RStudio established itself as the ‘go to’ data science IDE for R programming and was extended to allow integration of R code, narrative text, and figures into a single notebook interface using “RMarkdown” (Baumer et al. 2014). The software companies Enthought Inc. and Anaconda (formerly Continuum Analytics) independently developed distributions of the Python programming language to include core scientific computing packages. Anaconda later extended their distribution to include R. In 2015, the non-profit Project Jupyter was established (Kluyver et al. 2016) to “develop open-source software, open-standards, and services for interactive computing across dozens of programming languages” (Project Jupyter 2019). Their main product is Jupyter Notebook, a browser-based interactive data science notebook environment. Jupyter Notebook allows seamless integration of code, narrative text, and figures into a single *live* executable and editable document, recorded in the open-standard and language-independent JavaScript Object Notation (JSON) format. Notebooks may be written in a single programming language, or a combination of multiple languages. Jupyter Notebooks can use *kernels* for new or more specialised languages (such as Kotlin, GAP, Haskell, etc.), which gives them an advantage of being agnostic to programming language. Finally, integration of Jupyter Notebooks with the Docker (www.docker.com) virtualization platform enables operating system level working environments to be packaged into virtual “containers”, which allows collections of notebooks and the supporting third-party tools and software to be deployed as public, self-contained, reproducible interactive services using cloud computing.

### Collaboration through cloud computing

Open and dynamic collaboration on projects is critical to effective working but remains a significant challenge for researchers. There is a real and present need for efficient sharing and management of files that allows easy access, use, and version control (a documented history of the changes made to a file, enabling step-by-step reproduction and reconstruction of its development) for all collaborators. Widely-used collaboration mechanisms such as sharing code via email or online blogs are cumbersome, frequently leading to conflicts between the work of different researchers as they work on the same files at the same time in different locations. Cloud services including Box, Google Drive, and Dropbox have become essential tools for scientists by providing shared online data and document storage. Tools such as Microsoft Office Online and Google Suite provide real-time collaboration tools enabling true simultaneous editing of a single document by multiple authors, and services like Dropbox are able to track edits and prompt users to keep local copies of files up to date. Both approaches allow users to step back through document history as a rudimentary form of version control. They reduce practical barriers to collaborative working and reduce frustration and conflicts resulting from two or more people editing different copies of the same file at the same time. Collaborative working on metabolomic data analysis workflows would benefit from adoption of similar approaches.

The source code hosting facilities Bitbucket, GitHub and SourceForge are currently the dominant platforms for sharing and collaborating on (particularly open-source) software. GitHub has become the largest source code hosting facility in the world, with over 36 million users and 100 million repositories (GitHub 2019). These facilities offer many benefits including: free public (and private) source code repositories; enforced best practice through version control; and additional administrative and project management services that foster collaboration, including project webpages and wikis, issue tracking, code reviews, and task management. This makes GitHub and similar services a practical option for development, publication and distribution of Jupyter Notebooks, together with their associated source code and test data.

Services such as GitHub and BitBucket allow collaborators to view and edit static code and view static notebooks, but code cannot be executed directly on their servers. To run Jupyter Notebooks and associated source code, the user must either download and run a local copy of the files, or upload and run the notebook “in the cloud” using a cloud infrastructure provider such as Amazon Web Services, Google Colab, Openstack, or Microsoft Azure. The process of enabling the practical use of this shared resource can therefore require a level of computational expertise that may be a deterrent to casual users and restrict uptake by non-expert data-curious scientists.

The PhenoMeNal portal (http://phenomenal-h2020.eu) is an elegant solution to this problem for the metabolomics community. PhenoMeNal (Peters et al. 2019) is an easy-to-use, cloud-based metabolomics research environment led by EMBL’s European Bioinformatics Institute. The PhenoMeNal App Library includes over 50 widely used metabolomics data analysis tools that can be accessed either through Jupyter or Galaxy and deployed using a cloud infrastructure provider. This curated software library allows the community to maintain consistency across workflows but, in common with other GUI tools and centrally-managed workflow approaches, it can be restrictive.

A comparable but completely general public service is provided by the Binder team at mybinder.org (Project Jupyter et al. 2018). Binder is an open-source web service that allows users to share notebooks by creating a temporary cloud-based copy of the GitHub repository that contains them. This enables reproducible sharing of interactive and editable Jupyter or Rstudio notebooks as a virtual machine running in the cloud. The user can start and access a new virtual machine running live notebooks by following a single web link. In use, the notebooks appear to the user as if they were any other Jupyter notebook running on their own computer, with all the necessary dependencies, supplementary code and data pre-installed. Using the Binder framework gives researchers the power to reproduce and thoroughly test published results, or apply the analyses to their own data by running the source code interactively in their browser. In this tutorial review we take the reader through a process of using, writing, and deploying Jupyter Notebooks on Binder to help them take control of their own data science, and share their work through open science approaches.

## Experiential learning tutorials

**Fig. 2:**
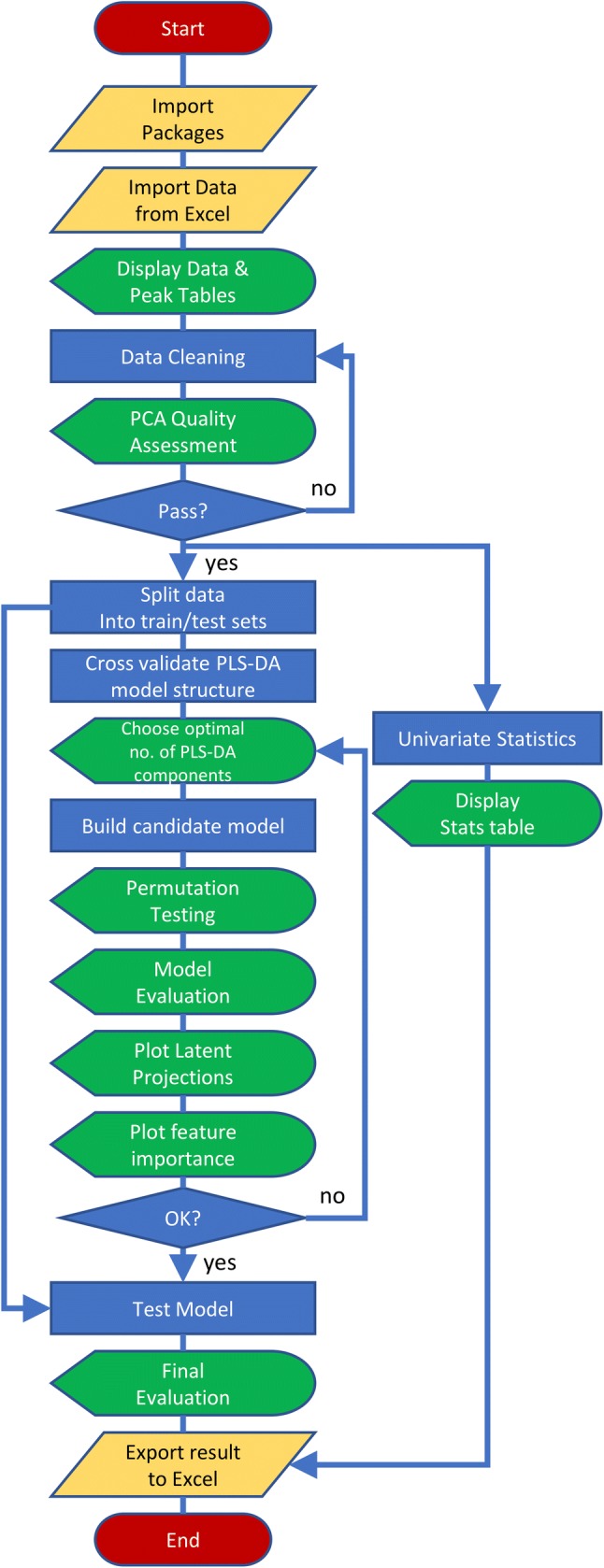
Metabolomics data analysis workflow. The workflow implemented in Tutorials 1 and 2 represents a typical metabolomics data science workflow for a binary classification outcome. The following steps are included: data import, data cleaning based on pooled QC relative standard deviation, PCA to visually inspect data reproducibility, univariate statistics, multivariate machine learning (PLS-DA including cross validation, feature selection, and permutation testing). The flow diagram is coloured by primary operation type (yellow = data import/export; green = data visualisation; blue = data processing)

The remainder of this review provides readers with an experiential learning opportunity (Kolb 1984) using an example interactive metabolomics data analysis workflow deployed using a combination of Python, Jupyter Notebooks, and Binder. We assume that the initial stage of data-processing for the computational workflow (converting raw instrument files into an annotated data table) has already been completed, and that a deconvolved, but not necessarily annotated, data table has been created and checked for errors. These assumptions are made to make the learning objectives presented manageable, not as a directive for obfuscating the complete metabolomics workflow. It is possible, and encouraged, to include all data processing steps in interactive notebooks. The tutorial takes the reader through the process of using interactive notebooks to produce a shareable, reproducible data analysis workflow that connects the study design to reported biological conclusions in an interactive document, using data from two previously published metabolomics studies. This workflow includes a discrete set of interactive and interlinked procedures: data cleaning, univariate statistics, multivariate machine learning, feature selection, and data visualisation (Fig. 2).

The following five tutorials have been pedagogically designed to lead the reader through increasing levels of cognitive complexity, according to Bloom’s revised taxonomy (Anderson et al. 2001):

1. Launch and walk through a published Jupyter notebook using Binder in the cloud to *duplicate* a set of results.
2. Interact with and edit the content of a published Jupyter notebook using Binder in the cloud to *understand* workflow methods.
3. Install Python and use published Jupyter Notebooks on the researcher’s computer to *apply* and *experiment* with workflow methods locally.
4. *Create* a metabolomics Jupyter notebook on a local computer.
5. Deploy the Jupyter notebook from Tutorial 4 on Binder in the cloud via GitHub.

### Overview of Jupyter/GitHub/Binders

**Fig. 3:**
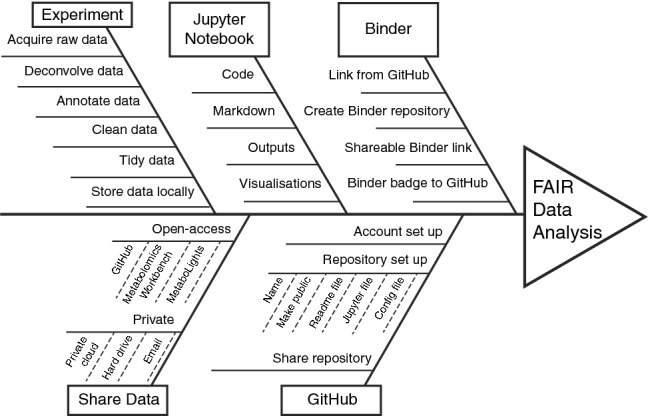
Key elements required for FAIR data analysis, using Jupyter Notebooks and Binder deployment. A fishbone diagram describing the detailed requirements for FAIR data analysis in metabolomics. Experimental data are derived from typical metabolomics workflows and formatted appropriately for analysis. Data need to be shared, either privately (for pre-publication collaboration) or publicly (for open dissemination). The Jupyter Notebook contains all code, markdown comments, outputs, and visualisations corresponding to the study. The Jupyter Notebook and other required files (such as Readme and configuration files) are compiled into a public GitHub repository. Finally, Binder is used to easily deploy and share the Jupyter Notebook

Before beginning the tutorial, we review some fundamental concepts behind *Jupyter Notebooks*, *GitHub*, and *Binder*, as understanding these can aid successful independent execution of this open-science approach (Fig. 3). All code embedded in each of the example notebooks is written in the Python programming language and is based upon extensions of popular open source packages with high levels of community uptake and support. These include: Numpy for matrix-based calculations (van der Walt et al. 2011); Pandas for high level data table manipulation (McKinney 2017); Scikit-learn for machine learning (Pedregosa et al. 2011); and Matplotlib (Hunter 2007), Bokeh (Bokeh Development Team 2018), Seaborn (Waskom et al. 2018), and BeakerX (Beaker X Development Team 2018) for data visualisation. Additionally, we deploy a simple package called ‘cimcb-lite’, developed by the authors for this publication, that integrates the functionality of the above packages into a set of basic methods specific to metabolomics. A tutorial on the Python programming language itself is beyond the scope of this publication, but we hope that the code presented is sufficiently well-documented in each notebook to be understood. Many excellent publications can be consulted for an in-depth introduction to using Python for data science (Jones 2013; Ramalho 2015; The Carpentries 2019; VanderPlas 2016).

Digital object identifiers (DOI) are widely used to identify academic and government information in the form of journal articles, research reports and data sets. It is also possible to assign a DOI to open access software. Specifically, researchers are able to make the work shared on GitHub citable by archiving with a data archiving tool such as Zenodo (www.zenodo.org) (Sicilia et al. 2017). A detailed tutorial is available (Open Science MOOC 2018). This archiving tool will ‘fix’ in time a given repository (e.g. Jupyter notebook and meta data), so that it can be associated with a particular static publication, while allowing the programmer to further develop the notebook on GitHub. The tutorials in this paper are archived with the handle 10.5281/zenodo.3362624 (10.5281/zenodo.3362624).

#### Jupyter Notebook

**Fig. 4:**
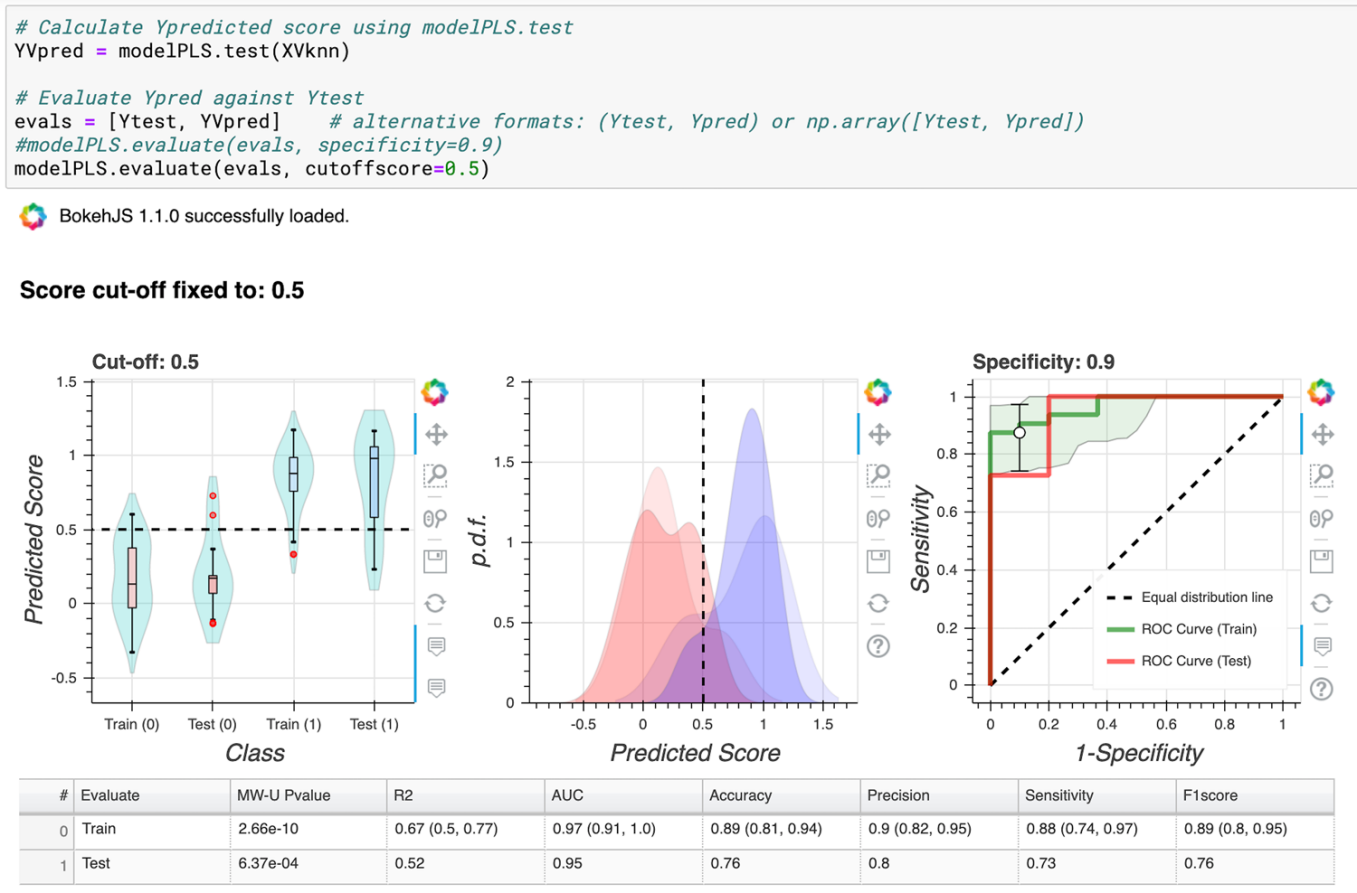
Example Jupyter Notebook Screenshot. At the top of the page, there is the Jupyter menu bar and ribbon of action buttons. The main body of the notebook then displays text and code cells, and any outputs from code execution. This screenshot taken near the end of Tutorial 1 when the partial least squares discriminant analysis model is being evaluated. Three plots are generated, showing comparisons of the performance of the model on training and holdout test datasets: a violin plot showing the distribution of known positive and negative in both training and test sets, and the class cut-off (dotted line); probability density functions for positive and negative classes in the training and test sets (the training set datapoints are rendered as more opaque); ROC curves of model performance on training (with 95% CI) and test set

Jupyter Notebook (jupyter.org) is a powerful, open-source, browser-based tool for interactive development and presentation of data science projects. Each notebook consists of a collection of executable cells, and each cell contains either text formatted using the Markdown language (Gruber 2004) or executable code (usually Python or R). When a ‘code cell’ is executed any graphical or text output (numerical results, figures or tables) is presented within the document immediately below the cell. Figure 4 shows an example of a notebook after execution. A popular way to get started with Jupyter Notebooks is to install the Anaconda distribution (anaconda.com), for which graphical installers are available on Windows, macOS and Linux operating systems (anaconda.com/distribution/). After installation a local Jupyter server can be launched using the Anaconda-Navigator application. To run a specific local Jupyter notebook with Anaconda-Navigator the user can navigate to the appropriate local folder using the browser-based interface, and click on the desired notebook file (which can be identified by the .*ipynb* suffix).

#### GitHub

GitHub (github.com) is a cloud-based web service that helps programmers store, manage, and share their code (and associated data files), as well as track and control changes to their code (version control). It is free to sign up and host a public code repository, which makes GitHub especially popular with open-source projects and a good choice for distributing Jupyter Notebooks, project-specific code and documentation. Jupyter Notebooks stored publicly on GitHub can be downloaded and run on a local machine using Anaconda or linked to a cloud-based platform. To complete all the steps of this tutorial a (free) GitHub account is required. An account at GitHub may be created by clicking “sign up” on the GitHub home page (github.com) and following the instructions.

#### Binder

Binder (mybinder.org) is an open source web service that allows users to deploy a GitHub repository comprising a collection of Jupyter Notebooks (with configuration files that describe the required computing environment) as a temporary cloud-based virtual machine. The Binder deployment is accessible by web browser and includes the programming language and all necessary packages and data. As with all publicly-accessible cloud storage care must be taken if data are sensitive or private. Researchers can launch the virtual machine in their browser but, because the user environment is temporary, once the session is closed all new results are lost. If changes are made, the user must download any changed files or output they wish to keep.

### Tutorials

#### Tutorial 1: launching and using a Jupyter Notebook on Binder

This tutorial demonstrates the use of computational notebooks for transparent dissemination of data analysis workflows and results. The tutorial steps though a metabolomics computational workflow implemented as a Jupyter Notebook and deployed on Binder. The workflow is designed to analyse a deconvolved and annotated metabolomics data set (provided in an Excel workbook) and is an example of the standard data science axiom: *Import, Tidy, Model,* and *Visualise*.

The Jupyter notebook for this tutorial is named *Tutorial1.ipynb* and is available at GitHub in the repository https://github.com/cimcb/MetabWorkflowTutorial. This repository can be downloaded (*cloned*) to the researcher’s own computer, or run on the Binder service. In the text we assume that the tutorial is being run using the Binder service. To open the notebook on Binder, go to the tutorial homepage: https://cimcb.github.io/MetabWorkflowTutorial and click on the topmost “Launch Binder” icon to “launch the tutorial environment in the cloud”. It will take a short while for Binder to build and deploy a new temporary virtual machine. Once this is ready the Jupyter notebook landing page will show the files present in this copy of the GitHub repository (Supplementary Fig. 1).

The tutorial workflow analysis interrogates a published dataset used to discriminate between samples from gastric cancer and healthy patients (Chan et al. 2016). The dataset is available in the Metabolomics Workbench database (http://www.metabolomicsworkbench.org, Project ID PR000699). For this tutorial, the data are stored in the Excel workbook *GastricCancer_NMR.xlsx* using the *Tidy Data* framework (Wickham 2014): each variable is a column, each observation is a row, and each type of observational unit is a table. The data are split into two linked tables. The first, named ‘Data’, contains data values related to each observation. i.e. metabolite concentrations M_1_ … M_n_, together with metadata such as: ‘sample type’, ‘sample identifier’ and ‘outcome class’. The second, named ‘Peak’, contains data that links each metabolite identifier (M_i_) to a specific annotation and optional metadata (e.g. mass, retention time, MSI identification level, number of missing values, quality control measures, etc.). The Excel file can also be downloaded from the Binder virtual machine for inspection on your own machine by selecting the checkbox next to the filename and clicking on the Download button in the top menu (Supplementary Fig. 1).

To begin the tutorial, click on the *Tutorial1.ipynb* filename (Supplementary Fig. 1). This will open a new tab in your browser presenting the Jupyter notebook (Supplementary Fig. 2). At the top of the page there is a menu bar and ribbon of action buttons similar to those found in other GUI-based software, such as Microsoft Word. The interface is powerful, and it is worth taking time to become familiar with it, but for this tutorial only the “Run” button and the “Cell” and “Kernel” drop down menus are required.

The rest of the page is divided into “code cells” and “text cells”. The “text cells” briefly outline the context and computation of the “code cells” beneath them. Code and text cells can be distinguished by their background colour (code cells are slightly grey, text cells are slightly red), by the text formatting (code cells have a fixed-width font, text cells have word processor-like formatting), and the “In []:” marker text is present next to each code cell.

To run a single code cell, first select it by clicking anywhere within the cell, which will then be outlined by a green box (if you select a text cell, this box is blue—Supplementary Fig. 3). Once a cell is selected, the code in the cell can be executed by clicking on the “Run” button in the top menu. Multiple cells can also be run in sequence by choosing options from the dropdown list in the “Cell” menu item. The options include “Run All” (runs all the cells in the notebook, from top to bottom), and “Run all below” (run all cells below the current selection). These can be used after changing the code or values in one cell to recalculate the contents of subsequent cells in the notebook.

The “computational engine” that executes the code contained in a notebook document is called the *kernel*, and it runs continually in the background while that notebook is active. When you run a code cell, that code is executed by the kernel and any output is returned back to the notebook to be displayed beneath the cell. The kernel stores the contents of variables, updating them as each cell is run. It is always possible to return to a “clean” state by choosing one of the “Restart Kernel” options from the “Kernel” menu item’s dropdown list. Selecting “Restart & Run All” from the “Kernel” dropdown menu will restart the kernel and run all cells in order from the start to the end of the notebook.

Beginning from a freshly-loaded *Tutorial1.ipynb* notebook in the Binder, clicking on “Cell->Run All” or “Kernel->Restart & Run All” will produce a fully executed notebook that matches the output in the static supplementary html file *Tutorial1.html* (cimcb.github.io/MetabWorkflowTutorial/Tutorial1.html). Choosing “Restart and Clear Outputs” from the “Kernel” dropdown menu, will reset the notebook and clear all data from memory and remove any outputs, restoring its original state.

The tutorial can be completed by reading the text cells in the notebook and inspecting, then running, the code in the corresponding code cells. This is an example of “Literate Programming” that weaves traditional computing source code together with a human-readable, natural language description of the program logic (Knuth 1984). The notebook interface makes notable advances on the original proposition for literate programming that are used in this tutorial, the most significant of which is that the output of running the code is also incorporated into the document. The browser interface allows for further enhancements, such as hyperlinks to external webpages for explanations and further reading about technical terms, embedded interactive spreadsheet-like representation of large datasets (e.g. section 2. Load Data and Peak Sheet), and embedded interactive graphical output (e.g. section 4. PCA Quality Assessment).

#### Tutorial 2: interacting with and editing a Jupyter Notebook on Binder

The second tutorial is interactive and showcases the utility of computational notebooks for both open collaboration and experiential education in metabolomics data science. Tutorial 2 is accessed on GitHub through the same process as described for Tutorial 1. To open the notebook on Binder, go to the tutorial homepage: https://cimcb.github.io/MetabWorkflowTutorial and click on the topmost “Launch Binder” icon to “launch the tutorial environment in the cloud”, then click the *Tutorial2.ipynb* link on the Jupyter landing page. This will present a new tab in your browser containing the second tutorial notebook. The functionality of this notebook is identical to Tutorial 1, but now the text cells have been expanded into a comprehensive interactive tutorial. Text cells, with a yellow background, provide the metabolomics context and describe the purpose of the code in the following code cell. Additional coloured text boxes are placed throughout the workflow to help novice users navigate and understand the interactive principles of a Jupyter Notebook:

##### Action (red background labelled with ‘gears’ icon)

Red boxes provide suggestions for changing the behaviour of the subsequent code cell by editing (or substituting) a line of code. For example, the first red cell describes how to change the input dataset by changing the path to the source Excel file.

##### Interaction (green background with ‘mouse’ icon)

Green boxes provide suggestions for interacting with the visual results generated by a code cell. For example, the first green box in the notebook describes how to sort and colour data in the embedded data tables.

##### Notes (blue background with ‘lightbulb’ icon)

Blue boxes provide further information about the theoretical reasoning behind the block of code or a given visualisation. This information is not essential to understand Jupyter Notebooks but may be of general educational utility and interest to new metabolomics data scientists.

To complete the tutorial, first execute the notebook by selecting the “Restart & Run All” option in the “Kernel” dropdown menu. Move through the notebook one cell at a time reading the text and executing the code cells. When prompted, complete one (or multiple) modifications suggested in each ‘action’ box, and the click “Run all below” from the “Cell” dropdown menu, observing the changes in cell output for all the subsequent cells. Further guidance is included in the notebook itself.

It is possible to save the edited notebook to the Binder environment, but any changes made to the notebook during the tutorial are lost when the Binder session ends. To keep changes made to the tutorial notebook or its output, modified files must be downloaded to your local computer before you end the session. Modified files can also be downloaded from the Jupyter landing page. To download files, click the checkbox next to each file you wish to download, and then click the ‘Download’ button from the top menu.

#### Tutorial 3: downloading and installing a Jupyter Notebook on a local machine

Jupyter Notebooks can be run on a standard laptop or desktop computer in a number of different ways, depending on the operating system. The Anaconda distribution provides a unified, platform-independent framework for running notebooks and managing Conda virtual environments that is consistent across multiple operating systems, so for convenience we will use the Anaconda interface in these tutorials.

To install the Anaconda distribution, first download the *Python 3.x Graphical Installer* package from the Anaconda webpage (https://www.anaconda.com/distribution/) then open the installer and follow the instructions to compete the installation (https://docs.anaconda.com/anaconda/install/). Be sure to download the installer package specific to your computer’s operating system (e.g. macOS, Microsoft Windows or Linux). When the process is completed, the “Anaconda Navigator” application will be installed in your applications folder.

To start Jupyter on your machine first launch the Anaconda Navigator application. This will display a home screen with a sidebar menu on the left-hand side and the main area showing a panel of application icons, with short descriptions. Locate the Jupyter Notebook application and icon in this panel and click the “launch” button under the icon. This will start a Jupyter web server and open the Jupyter landing page in your default web browser. To run an existing Jupyter notebook, navigate to the appropriate folder on your computer’s filesystem in the Jupyter landing page, and click on the notebook (.ipynb) file you wish to open. To end a Jupyter session, click on the “quit” button in the top right-hand corner of the Jupyter landing page. Quit now if you have been working along.

To run the Tutorial notebooks, we need to download the tutorial repository containing those notebooks from GitHub and set up a local “virtual environment” that contains the programming libraries and software tools necessary to run the code cells in the notebooks.

To download the notebook and associated files from the Github repository page (https://github.com/cimcb/MetabWorkflowTutorial), click on the green button labelled “clone or download” and choose the option to “Download ZIP”. Save the zip file (MetabWorkflowTutorial-master.zip) in a convenient location. Extract the zip file to create a new folder in the same location as the .zip file, called “MetabWorkflowTutorial-master”. The contents of this folder are the files visible in the repository at the GitHub site. We will refer to this folder as the “repository root”, or just “root”.

The Jupyter Notebooks in the repository require several Python packages to be installed in order to be run successfully. It would be possible to install these on the local computer so that they are visible to, and accessible by, all notebooks on the computer. However, it is often the case that different repositories and projects require alternative, incompatible versions of these packages. So, in practice, it is not usually possible to install a single set of packages that meets the needs of all the projects that a user would want to run. A technical solution to this is to create a new “virtual environment” that contains only the packages necessary for a project to run, and keeps them separate (“sandboxes” them) from any other projects. Environments can be created when required, and deleted when no longer necessary, without affecting other projects or the operation of the computer. It is good practice to create a new virtual environment for each project, and typical that multiple such environments are set up, and exist simultaneously on the same computer. The Anaconda Navigator application provides an interface for creating and managing these virtual environments.

To create a new virtual environment for the tutorial, first open the Anaconda Navigator application and click on “Environments” in the left-hand sidebar. The main panel will change to list any *virtual environments* that have been created using Anaconda. If no environments have been created only “base (root)” will be listed. To the right of each virtual environment Anaconda Navigator lists the packages that have been installed in that environment.

It is common to create a new environment “from scratch” by specifying individual packages in the Anaconda Navigator, but for this tutorial we will use a configuration file called “environment.yml” that is part of the GitHub repository. This file describes all the packages that are necessary to reproduce an environment for running the tutorial notebooks. To create a new environment from this configuration file, click on “Import” (at the bottom of the main panel of Anaconda Navigator) and navigate to the repository root folder. By default Anaconda Navigator expects configuration files with “.yaml” or “.yml” file extensions, so only the file named “environment.yml” should be highlighted in the file dialog box. Select this file and click “Open”. The “Import new environment” dialogue box will have autocompleted the “Name:” field for the new environment (“MetabWorkflowTutorial”). To complete creation of the new environment, click on the “Import” button. Anaconda Navigator will show a progress bar in the main panel as it creates the new environment.

Once the environment has been created, click on the “Home” icon in the left-hand sidebar. In the main panel, the dropdown should now read “Applications on [MetabWorkflowTutorial]”, which indicates that the MetabWorkflowTutorial environment which was just created is now active. If “MetabWorkflowTutorial” is not visible, click on the dropdown menu and select that environment. Click on the “Launch” button under Jupyter Notebook in the main panel, to launch Jupyter in your web browser.

The Jupyter landing page will start in your home folder. To use the tutorial notebooks, navigate to the repository root. The notebooks for Tutorial 1 and 2 can now be run on your own computer, just as on Binder, by selecting the appropriate notebook file. However any output or changes to the contents of a notebook file will now be saved persistently in the local computer and can be reused at any time.

As an alternative you may wish to try to create a virtual environment and launch Jupyter in your web browser through a terminal window (command window). To do this open the terminal window (type ‘*terminal’* in your computer’s search box), then type the following five lines of code:

- git clone https://github.com/cimcb/MetabWorkflowTutorial
- cd MetabWorkflowTutorial
- conda env create -f environment.yml
- conda activate MetabWorkflowTutorial
- jupyter notebook

Line one creates an exact copy of the github file directory on your local machine in the folder ‘MetabWorkflowTutorial’. Line two moves you into that folder. Line three creates the virtual environment called “MetabWorkflowTutorial” using the contents of the environment.yml file. Line four activates the virtual environment. Line five launches a local Jupyter notebook server and opens the Jupyter landing page in your web browser, from which you can run the tutorials.

To close the local Jupyter notebook server press “<control>c” *twice* in the terminal window and it will ask you to confirm the action. You may then close the virtual environment by typing:

- conda deactivate

When you no longer need the virtual environment, the following will delete it from your computer:

- conda remove –name MetabWorkflowTutorial –all

If you created a virtual environment using Anaconda Navigator you will have to delete the environment before creating a fresh version.

#### Tutorial 4: creating a new Jupyter Notebook on a local computer

Tutorial 4 builds on tutorial 3. Please ensure that the Anaconda Python distribution is installed on your computer.

In this tutorial we will create a new Jupyter notebook that demonstrates the use of visualisation methods available in Anaconda Python without the need to install additional third-party packages. We will upload a generic metabolomics data set and write code to produce four graphical outputs:

1. A histogram of the distribution of QC_RSD_ across the data set.
2. A kernel density plot of QC_RSD_ vs. D-ratio across the data set.
3. A PCA scores plot of the data set labelled by sample type.
4. A bubble scatter plot of molecular mass vs. retention time, with bubble size proportional to QC_RSD_

The data set included in this tutorial is previously unpublished, and of arbitrary biological value. It describes serum data acquired using a C18+ LC–MS platform consisting of 3084 unidentified peaks and 91 samples. Of the 91 samples, 23 are pooled QCs injected every 5th sample across the experimental run. The Peak table contains information on the molecular mass, retention time of each detected metabolite, and the associated QCRSD and D-ratio values calculated following recommended quality control procedures (Broadhurst et al. 2018). The data are presented in an Excel file using the previously-described “tidy data” format.

Tutorial 4 is available in a GitHub repository at https://github.com/cimcb/MetabSimpleQcViz. Download and unzip the repository to a folder on your own computer, using the method described in Tutorial 3 (the location of this folder will now be the “*repository root*”). This copy (clone) of the repository is for reference only as we will be recreating the contents of this directory under a different name as we move through this tutorial and Tutorial 5.

First create a new Jupyter notebook. To do this, start the Anaconda Navigator application if it is not already open. Ensure that “[base (root)]” is selected in the “Applications on” dropdown list of the main panel, then launch Jupyter Notebook. This will start a new Jupyter notebook server in your browser and show files from the home directory on the landing page. Navigate to the repository root (the “MetabSimpleQcViz” folder). To create a new notebook, click on the “New” button in the top right corner of the page. This will list supported Jupyter Notebook languages in the dropdown. Select “Python 3” from this list. A new tab will open in your browser, showing a blank notebook called “Untitled” (at the top of the page). Rename the notebook by clicking on the text “Untitled” and replacing it with “myExample”. This will create a new file in the repository called “myExample.ipynb”

When the “myExample.ipynb” notebook is launched, it contains a single empty code cell. We will use this cell to add a title to the notebook. To do this we need to convert the cell type to be a Markdown cell, then type a header in the cell, and execute it. First, select the empty cell by clicking anywhere within the cell. To convert the cell type, click on the dropdown field marked “Code” in the top menu bar and select “Markdown”. The “In[]:” prompt should disappear from the left-hand side of the cell. Now click inside the cell to see the flashing cursor that indicates the cell is ready to accept input. Type “# Tutorial 4” and click on the “Run” button in the top menu. The formatting of the first cell should change, and a new code cell should appear beneath it.

In the new code cell, we will place Python code that:

1. Imports the Pandas package (necessary to load the Excel spreadsheet).
2. Loads the dataset into variables called “data” and “peak”.
3. Report the number of rows and column in the tables.
4. Displays the first few lines of the resulting table.

The required code is provided in the static supplementary html file Tutorial4*.html* (https://cimcb.github.io/MetabSimpleQcViz/Tutorial4.html) and “Tutorial4.ipynb” notebook and can be copy-and-pasted or typed in manually, as preferred. When the code is complete, click on the “Run” button again to execute the cell. On completion, two tables should be visible below the code cell (one for “data”, one for “peak”), and a new empty code cell should be placed beneath this.

Next we add the code required to draw a histogram of the RSD values across all the detected peaks in this data set. Using the Tutorial4.html file as a guide, add in the required explanatory text and Python code and click on the “Run” button after each step.

Continue adding in the remaining explanatory text and Python code using the Tutorial4.html file. After completion you will have a Jupyter notebook that takes a metabolomics dataset through the process of generating diagnostic plots for quality control. Once you are satisfied with the state of the notebook, it can be saved by clicking on the floppy disk icon (far left on the menu). The notebook can then be closed by clicking “File” and then “Close and Halt” from the top Jupyter menu. The notebook tab will be closed, showing the Jupyter landing page. The Jupyter session can be closed by clicking on “Quit” on the Jupyter landing page tab of your web browser (this tab may not close automatically).

#### Tutorial 5: deploying a Jupyter Notebook on Binder via GitHub

Tutorial 5 builds on tutorial 3 and 4. To complete this tutorial, we will create a new GitHub repository. A GitHub account is required for this. If you do not already have a GitHub account, please follow the instructions on GitHub at https://help.github.com/en/articles/signing-up-for-a-new-github-account.

To create a new repository, log into the GitHub site (if you are not already logged in) and navigate to your profile page (https://github.com/<yourusername>), then click on the “Repositories” link at the top of the page. To start a new repository, click on the “New” button at the top right of the page. This will open a new page titled “Create a new repository.” Each repository requires a name, and this should be entered into the “Repository name” field; use the name “JupyterExample”. Beneath the Repository Name field there is an optional Description box, and then below this a choice of public or private repository. Ensure that the ‘Public’ option is chosen. Select the checkbox to “Initialize this repository with a README” (this is a file in which you will write useful information about the repository, later). Below this is the option to “Add a license” file. There are many alternative licences to choose from (https://choosealicense.com/), and the choice for your own projects may be constrained by funder, home organisation, or other legal considerations. We strongly recommend that all projects carry a suitable licence, and that you add the MIT License to this tutorial repository. Now, to create the repository, click the “Create repository” button.

On successful creation of the repository, GitHub will present the new repository’s home page (this will be at https://github.com/<yourusername>/JupyterExample), with some options for “Quick setup”. Under the “Quick setup” notice, the LICENSE and README.md file will be shown, and clicking on either will open them. The README.md file for a repository is automatically displayed on the homepage, but in this case, it is empty (we can add text later).

Now we need to add the new Jupyter notebook and the Excel data file from tutorial 4 to the repository. We will do this using the GitHub “Upload files” interface, though there are several other ways to perform this action. To use the GitHub interface, click on the ‘Upload files’ button and either drag files from your computer, or click on “choose your files” to select files with a file dialogue box. Add the ‘myExample.ipynb’ and ‘data.xlsx’ files from your repository root. These files will be placed in the “staging area”, visible on the webpage but not yet committed to the repository.

GitHub imposes version control as a form of best practice on the repositories it hosts. One of the features of version control best practice is that a description of the changes made to a repository should accompany every “commit” to that repository. To do this, enter the text “Add data and notebook via upload” to the top field under “Commit changes.” Then, to commit the files to the repository, click on the “Commit changes” button.

Now that there is a publicly hosted GitHub repository containing a notebook and dataset, we are nearly ready to make the notebook available interactively through Binder. The final necessary component required is a configuration file. This file is vital, as it defines the environment Binder will build, with a specified programming language and all the necessary packages for the notebook to successfully operate. This configuration file is an Anaconda YAML file called ‘*environment.yml*’ and it contains a list of *dependencies* (the programming language version and a list of packages used in the notebook) and *channels* (the location of these resources in the Anaconda cloud library). Detailed consideration of how to create these files is beyond the scope of the tutorial. Upload the environment.yml file from Tutorial 4 (it is also included in the Supplementary File, to cut and paste if required) to the repository in the same way that the notebook and data files were uploaded.

We are now ready to build and launch a Binder virtual machine for this repository. To do this, open https://mybinder.org in a modern web browser. The landing page presents a set of fields to be completed for Binder to build a virtual machine. The minimal requirement is to specify a GitHub repository URL in the “GitHub repository name or URL” field Enter the path to the home page of your repository (https://github.com/<yourusername>/JupyterExample) in this field, and click on the ‘Launch’ button. Binder will use the configuration file in the root directory to build and store a Docker image for your repository. This process often takes several minutes.

Once the Binder repository is built, the URL shown in the field “Copy the URL below and share your Binder with others” (here: https://mybinder.org/v2/gh/<yourusername>/JupyterExample/master) can be shared with colleagues. A button to launch the Binder can also be added into the README file on GitHub (we also strongly recommend this). Anyone using this URL in their browser, will be provided with an individual interactive session (1 CPU, 2 GB RAM running on Google Cloud) making available the notebooks of your repository in an interactive and editable form.

Congratulations, you have created your first Binder notebook! Now share it with your colleagues!

It is important to remind users that data uploaded to a public GitHub repository is indeed public. If the user wants to share Jupyter Notebooks but not any associated metabolomics data (or other sensitive data) then clear instructions on how to securely access and download the data needs to be included in the notebook text, and the location of that downloaded data be included in the requisite notebook code block (this could be a local hard drive, or uploaded to Binder while in session). If institutional security concerns preclude using a collaborative workspace such as Binder, then alternative cloud solutions such as Microsoft Azure can be investigated. Before doing so it is probably best that to consult with your institute IT representative.

## Summary

Due to the rate at which data are generated and new analysis and visualisation methods are developed, the omics sciences have become highly vulnerable to irreproducibility. In attempt to ameliorate this, the metabolomics community has made several efforts to align with FAIR data standards in the areas of open data formats, data repositories, online spectral libraries, and metabolite databases. While there are also a number of open options for data analysis, these tend to exist as prescriptive and inflexible workflows that inadvertently enable users to apply data science methods without fully understanding their underlying principles and assumptions. For FAIR data science to exist in metabolomics, presentation of methods and results needs to be rapid, transparent, reusable, and recoverably attached to published work. Furthermore, any framework enabling this must be intuitive and accessible to computational novices.

In this tutorial review, we have illustrated one possible solution for achieving open, transparent, yet intuitive data science within the metabolomics community. *Jupyter Notebooks* are an open-source, interactive web tool for creating seamless integration of text, code, and outputs (tables, figures) into a single live executable document. When used alongside data repositories, such as GitHub, and open cloud-based deployment services, such as Binder, these computational notebooks can greatly enhance transparent dissemination of data science methods and results during the publication process. In addition to the benefit of increased transparency, computational notebooks provide a valuable tool for open collaboration. Rather than exchanging multiple individual data, code, methods, and results files, computational notebook environments provide a single mechanism for collaborators (both within and beyond a single research group) to share and interact with the data science workflow. Moreover, this interactive nature, combined with the ability to provide extensive documentation, provides a valuable opportunity for enhanced learning in the computer programming and data science contexts. Given that they are increasingly recognised as being foundational to contemporary research, it is imperative that scientists continue to enhance these skills over the duration their career. This open and interactive framework enables scientists to continue to learn and also keep up-to-date with latest data science methods and trends without reinstalling the wheel.

## Electronic supplementary material

Below is the link to the electronic supplementary material.

Supplementary material 1 (DOCX 1514 kb)

Supplementary material 2 (HTML 764 kb)

Supplementary material 3 (HTML 760 kb)

Supplementary material 4 (HTML 647 kb)

## Data Availability

The metabolomics and metadata used in this paper were retrieved from Metabolights (https://www.ebi.ac.uk/metabolights/) study identifier: MTBLS290, and Metabolomics Workbench (https://www.metabolomicsworkbench.org/) project id: PR000699. This data were converted from the original data format to a clean format compliant with the Tidy Data framework, this is available at the CIMCB GitHub project page (https://github.com/CIMCB). All software developed for this paper is available at the CIMCB GitHub project page (https://github.com/CIMCB).

## References

[CR1] Afgan E, Baker D, Batut B, van den Beek M, Bouvier D, Cech M, Chilton J, Clements D, Coraor N, Gruning BA, Guerler A, Hillman-Jackson J, Hiltemann S, Jalili V, Rasche H, Soranzo N, Goecks J, Taylor J, Nekrutenko A, Blankenberg D (2018). The Galaxy platform for accessible, reproducible and collaborative biomedical analyses: 2018 update. Nucleic Acids Research.

[CR2] Anderson LW, Krathwohl DR, Airasian PW, Cruikshank KA, Mayer RE, Pintrich PR, Raths J, Wittrock MC (2001). A taxonomy for learning, teaching, and assessing: A revision of Bloom’s taxonomy of educational objectives.

[CR3] Baker M (2016). 1,500 scientists lift the lid on reproducibility. Nature.

[CR4] Baumer, B., Cetinkaya-Rundel, M., Bray, A., Loi, L. and Horton, N.J. (2014) R markdown: Integrating a reproducible analysis tool into introductory statistics, *Technology Innovations in Statistics Education, 8*

[CR5] Beaker X Development Team (2018). Beaker X. Retrieved May 1, 2019, from http://beakerx.com/.

[CR6] Bokeh Development Team (2018). Bokeh: Python library for interactive visualization. Retrieved May 1, 2019, from http://www.bokeh.pydata.org.

[CR7] Broadhurst D, Goodacre R, Reinke SN, Kuligowski J, Wilson ID, Lewis MR, Dunn WB (2018). Guidelines and considerations for the use of system suitability and quality control samples in mass spectrometry assays applied in untargeted clinical metabolomic studies. Metabolomics.

[CR8] Broadhurst DI, Kell DB (2006). Statistical strategies for avoiding false discoveries in metabolomics and related experiments. Metabolomics.

[CR9] Chan AW, Mercier P, Schiller D, Bailey R, Robbins S, Eurich DT, Sawyer MB, Broadhurst D (2016). (1)H-NMR urinary metabolomic profiling for diagnosis of gastric cancer. British Journal of Cancer.

[CR10] Considine EC, Thomas G, Boulesteix AL, Khashan AS, Kenny LC (2017). Critical review of reporting of the data analysis step in metabolomics. Metabolomics.

[CR11] Davidson RL, Weber RJM, Liu H, Sharma-Oates A, Viant MR (2016). Galaxy-M: A Galaxy workflow for processing and analyzing direct infusion and liquid chromatography mass spectrometry-based metabolomics data. GigaScience.

[CR12] Gehlenborg N, O’Donoghue SI, Baliga NS, Goesmann A, Hibbs MA, Kitano H, Kohlbacher O, Neuweger H, Schneider R, Tenenbaum D, Gavin AC (2010). Visualization of omics data for systems biology. Nature Methods.

[CR13] Giacomoni F, Le Corguillé G, Monsoor M, Landi M, Pericard P, Pétéra M, Duperier C, Tremblay-Franco M, Martin J-F, Jacob D, Goulitquer S, Thévenot EA, Caron C (2015). Workflow4Metabolomics: A collaborative research infrastructure for computational metabolomics. Bioinformatics (Oxford, England).

[CR14] GitHub (2019). About GitHub. Retrieved April 30, 2019, from https://github.com/about.

[CR15] Goodacre R, Broadhurst D, Smilde AK, Kristal BS, Baker JD, Beger R, Bessant C, Connor S, Capuani G, Craig A, Ebbels T, Kell DB, Manetti C, Newton J, Paternostro G, Somorjai R, Sjöström M, Trygg J, Wulfert F (2007). Proposed minimum reporting standards for data analysis in metabolomics. Metabolomics.

[CR16] Gruber, J. (2004). Markdown. Retrieved April 30, 2019, from https://daringfireball.net/projects/markdown/.

[CR17] Haug K, Salek RM, Conesa P, Hastings J, de Matos P, Rijnbeek M, Mahendraker T, Williams M, Neumann S, Rocca-Serra P, Maguire E, González-Beltrán A, Sansone S-A, Griffin JL, Steinbeck C (2012). MetaboLights—An open-access general-purpose repository for metabolomics studies and associated meta-data. Nucleic Acids Research.

[CR18] Holten D (2006). Hierarchical edge bundles: Visualization of adjacency relations in hierarchical data. IEEE Transactions on Visualization and Computer Graphics.

[CR19] Horai H, Arita M, Kanaya S, Nihei Y, Ikeda T, Suwa K, Ojima Y, Tanaka K, Tanaka S, Aoshima K, Oda Y, Kakazu Y, Kusano M, Tohge T, Matsuda F, Sawada Y, Hirai MY, Nakanishi H, Ikeda K, Akimoto N, Maoka T, Takahashi H, Ara T, Sakurai N, Suzuki H, Shibata D, Neumann S, Iida T, Tanaka K, Funatsu K, Matsuura F, Soga T, Taguchi R, Saito K, Nishioka T (2010). MassBank: A public repository for sharing mass spectral data for life sciences. Journal of Mass Spectrometry.

[CR20] Hunter JD (2007). Matplotlib: A 2D graphics environment. Computing in Science & Engineering.

[CR21] Jones M (2013). Python for biologists.

[CR22] Kluyver T, Ragan-Kelley B, Pérez F, Granger B, Bussonnier M, Frederic J, Kelley K, Hamrick J, Grout J, Corlay S, Ivanov P, Avila D, Abdalla S, Willing C, Birgi FAS (2016). Jupyter Notebooks—a publishing format for reproducible computational workflows in Loizides. Positioning and power in academic publishing: Players, agents and agendas.

[CR23] Knuth DE (1984). Literate programming. The Computer Journal.

[CR24] Kolb D (1984). Experiential learning: Experience as the source of learning and development.

[CR25] Lantz B (2013). Machine learning with R.

[CR26] Lee AH, Shannon CP, Amenyogbe N, Bennike TB, Diray-Arce J, Idoko OT, Gill EE, Ben-Othman R, Pomat WS, van Haren SD, Cao KL, Cox M, Darboe A, Falsafi R, Ferrari D, Harbeson DJ, He D, Bing C, Hinshaw SJ, Ndure J, Njie-Jobe J, Pettengill MA, Richmond PC, Ford R, Saleu G, Masiria G, Matlam JP, Kirarock W, Roberts E, Malek M, Sanchez-Schmitz G, Singh A, Angelidou A, Smolen KK, Consortium E, Brinkman RR, Ozonoff A, Hancock REW, van den Biggelaar AHJ, Steen H, Tebbutt SJ, Kampmann B, Levy O, Kollmann TR (2019). Dynamic molecular changes during the first week of human life follow a robust developmental trajectory. Nature Communications.

[CR27] McKinney W (2017). Python for data analysis.

[CR28] Müller AC, Guido S (2017). Introduction to machine learning with Python: A guide for data scientists.

[CR29] Open Science MOOC. (2018). Make your code citable using GitHub and Zenodo: A how-to guide. Retrieved August 14, 2019, from https://genr.eu/wp/cite/.

[CR30] Passey D (2017). Computer science (CS) in the compulsory education curriculum: Implications for future research. Education and Information Technologies.

[CR31] Pedregosa AF, Varoquaux AG, Gramfort AA, Michel AV, Thirion AB, Grisel AO, Blondel AM, Prettenhofer AP, Weiss AR, Dubourg AV, Vanderplas AJ, Passos AA, Cournapeau AD, Brucher AM, Perrot AM, Duchesnay AÉ (2011). Scikit-learn: Machine learning in Python. Journal of Machine Learning Research.

[CR32] Pedrioli PG, Eng JK, Hubley R, Vogelzang M, Deutsch EW, Raught B, Pratt B, Nilsson E, Angeletti RH, Apweiler R, Cheung K, Costello CE, Hermjakob H, Huang S, Julian RK, Kapp E, McComb ME, Oliver SG, Omenn G, Paton NW, Simpson R, Smith R, Taylor CF, Zhu W, Aebersold R (2004). A common open representation of mass spectrometry data and its application to proteomics research. Nature Biotechnology.

[CR33] Peters K, Bradbury J, Bergmann S, Capuccini M, Cascante M, de Atauri P, Ebbels TMD, Foguet C, Glen R, Gonzalez-Beltran A, Günther UL, Handakas E, Hankemeier T, Haug K, Herman S, Holub P, Izzo M, Jacob D, Johnson D, Jourdan F, Kale N, Karaman I, Khalili B, Emami Khonsari P, Kultima K, Lampa S, Larsson A, Ludwig C, Moreno P, Neumann S, Novella JA, O’Donovan C, Pearce JTM, Peluso A, Piras ME, Pireddu L, Reed MAC, Rocca-Serra P, Roger P, Rosato A, Rueedi R, Ruttkies C, Sadawi N, Salek RM, Sansone S-A, Selivanov V, Spjuth O, Schober D, Thévenot EA, Tomasoni M, van Rijswijk M, van Vliet M, Viant MR, Weber RJM, Zanetti G, Steinbeck C (2019). PhenoMeNal: processing and analysis of metabolomics data in the cloud. GigaScience.

[CR34] Pinu FR, Beale DJ, Paten AM, Kouremenos K, Swarup S, Schirra HJ, Wishart D (2019). Systems biology and multi-omics integration: Viewpoints from the metabolomics research community. Metabolites.

[CR35] Project Jupyter (2019). Jupyter. Retrieved March 19, 2019, from https://jupyter.org/.

[CR36] Project Jupyter, Bussonnier, M., Forde, J., Freeman, J., Granger, B., Head, T., Holdgraf, C., Kelley, K., Nalvarte, G., Osheroff, A., Pacer, M., Panda, Y., Perez, F., Ragan-Kelley, B. and Willing, C. (2018) Binder 2.0—Reproducible, interactive, sharable environments for science at scale, *SCIPY 2018*, *Proceedings of the 17th Python in Science Conference*, pp. 113–120.

[CR37] Ramalho L (2015). Fluent python: Clear, concise, and effective programming.

[CR38] Reinke SN, Galindo-Prieto B, Skotare T, Broadhurst DI, Singhania A, Horowitz D, Djukanović R, Hinks TSC, Geladi P, Trygg J, Wheelock CE (2018). OnPLS-based multi-block data integration: A multivariate approach to interrogating biological interactions in asthma. Analytical Chemistry.

[CR39] Rohart F, Gautier B, Singh A, Lê Cao K-A (2017). mixOmics: An R package for ‘omics feature selection and multiple data integration. PLoS Computational Biology.

[CR40] Sicilia M-A, García-Barriocanal E, Sánchez-Alonso S (2017). Community curation in open dataset repositories: Insights from Zenodo. Procedia Computer Science.

[CR41] Smith CA, O’Maille G, Want EJ, Qin C, Trauger SA, Brandon TR, Custodio DE, Abagyan R, Siuzdak G (2005). METLIN: A metabolite mass spectral database. Therapeutic Drug Monitoring.

[CR42] Spicer RA, Salek R, Steinbeck C (2017). A decade after the metabolomics standards initiative it’s time for a revision. Scientific Data.

[CR43] Sud M, Fahy E, Cotter D, Azam K, Vadivelu I, Burant C, Edison A, Fiehn O, Higashi R, Nair KS, Sumner S, Subramaniam S (2016). Metabolomics Workbench: An international repository for metabolomics data and metadata, metabolite standards, protocols, tutorials and training, and analysis tools. Nucleic Acids Research.

[CR44] Teschendorff AE (2019). Avoiding common pitfalls in machine learning omic data science. Nature Materials.

[CR45] The Carpentries (2019). Lessons. Retrieved May 20, 2019, from https://software-carpentry.org/lessons/.

[CR46] van der Walt S, Colbert SC, Varoquaux G (2011). The NumPy array: A structure for efficient numerical computation. Computing in Science & Engineering.

[CR47] VanderPlas J (2016). Python data science handbook: Essential tools for working with data.

[CR48] Waskom, M., Botvinnik, O., O’Kane, D., Hobson, P., Ostblom, J., Lukauskas, S., Gemperline, D.C., Augspurger, T., Halchenko, Y., Cole, J.B., Warmenhoven, J., Ruiter, J.d., Pye, C., Hoyer, S., Vanderplas, J., Villalba, S., Kunter, G., Quintero, E., Bachant, P., Martin, M., Meyer, K., Miles, A., Ram, Y., Brunner, T., Yarkoni, T., Williams, M.L., Evans, C., Fitzgerald, C., Brian and Qalieh, A. (2018). mwaskom/seaborn: v0.9.0. Retrieved May 1, 2019, from 10.5281/zenodo.1313201.

[CR49] Wickham H (2014). Tidy data. Journal of Statistical Software.

[CR50] Wilkinson MD, Dumontier M, Aalbersberg IJ, Appleton G, Axton M, Baak A, Blomberg N, Boiten J-W, da Silva Santos LB, Bourne PE, Bouwman J, Brookes AJ, Clark T, Crosas M, Dillo I, Dumon O, Edmunds S, Evelo CT, Finkers R, Gonzalez-Beltran A, Gray AJG, Groth P, Goble C, Grethe JS, Heringa J, ’t Hoen PAC, Hooft R, Kuhn T, Kok R, Kok J, Lusher SJ, Martone ME, Mons A, Packer AL, Persson B, Rocca-Serra P, Roos M, van Schaik R, Sansone S-A, Schultes E, Sengstag T, Slater T, Strawn G, Swertz MA, Thompson M, van der Lei J, van Mulligen E, Velterop J, Waagmeester A, Wittenburg P, Wolstencroft K, Zhao J, Mons B (2016). The FAIR guiding principles for scientific data management and stewardship. Scientific Data.

[CR51] Wishart DS, Feunang YD, Marcu A, Guo AC, Liang K, Vazquez-Fresno R, Sajed T, Johnson D, Li C, Karu N, Sayeeda Z, Lo E, Assempour N, Berjanskii M, Singhal S, Arndt D, Liang Y, Badran H, Grant J, Serra-Cayuela A, Liu Y, Mandal R, Neveu V, Pon A, Knox C, Wilson M, Manach C, Scalbert A (2018). HMDB 4.0: The human metabolome database for 2018. Nucleic Acids Research.

[CR52] Xia J, Broadhurst DI, Wilson M, Wishart DS (2013). Translational biomarker discovery in clinical metabolomics: An introductory tutorial. Metabolomics.

[CR53] Xia J, Wishart DS (2011). Metabolomic data processing, analysis, and interpretation using MetaboAnalyst. Current Protocols in Bioinformatics.

