# Supplementary material for: Toward collaborative open data science in metabolomics using Jupyter Notebooks and cloud computing
Source: Metabolomics. 2019 Sep 14;15(10):125. doi: 10.1007/s11306-019-1588-0 (PMC6745024; doi:10.1007/s11306-019-1588-0)
Supplement: Supplementary file 1 — Supplementary material 1 (DOCX 1514 kb) [file 11306_2019_1588_MOESM1_ESM.docx]

Toward Collaborative Open Data Science in Metabolomics using Jupyter Notebooks and Cloud Computing

**Authors**

Kevin M Mendez^1^, Leighton Pritchard^2^, Stacey N Reinke^1*^, David I Broadhurst^1*^

^1^Centre for Metabolomics & Computational Biology, School of Science, Edith Cowan University, Joondalup, 6027 Australia

^2^Information and Computational Sciences, James Hutton Institute, Invergowrie, Dundee DD2 5DA, Scotland

*Corresponding authors:

email: d.broadhurst@ecu.edu.au, stacey.n.reinke@ecu.edu.au

phone: +61 (0)8-6304-2705

List of supplementary html files:

1. Tutorial 1 : Tutorial1.html
2. Tutorial 2 : Tutorial2.html
3. Tutorial 4 : Tutorial4.html

Supplementary figures: pages 2-4


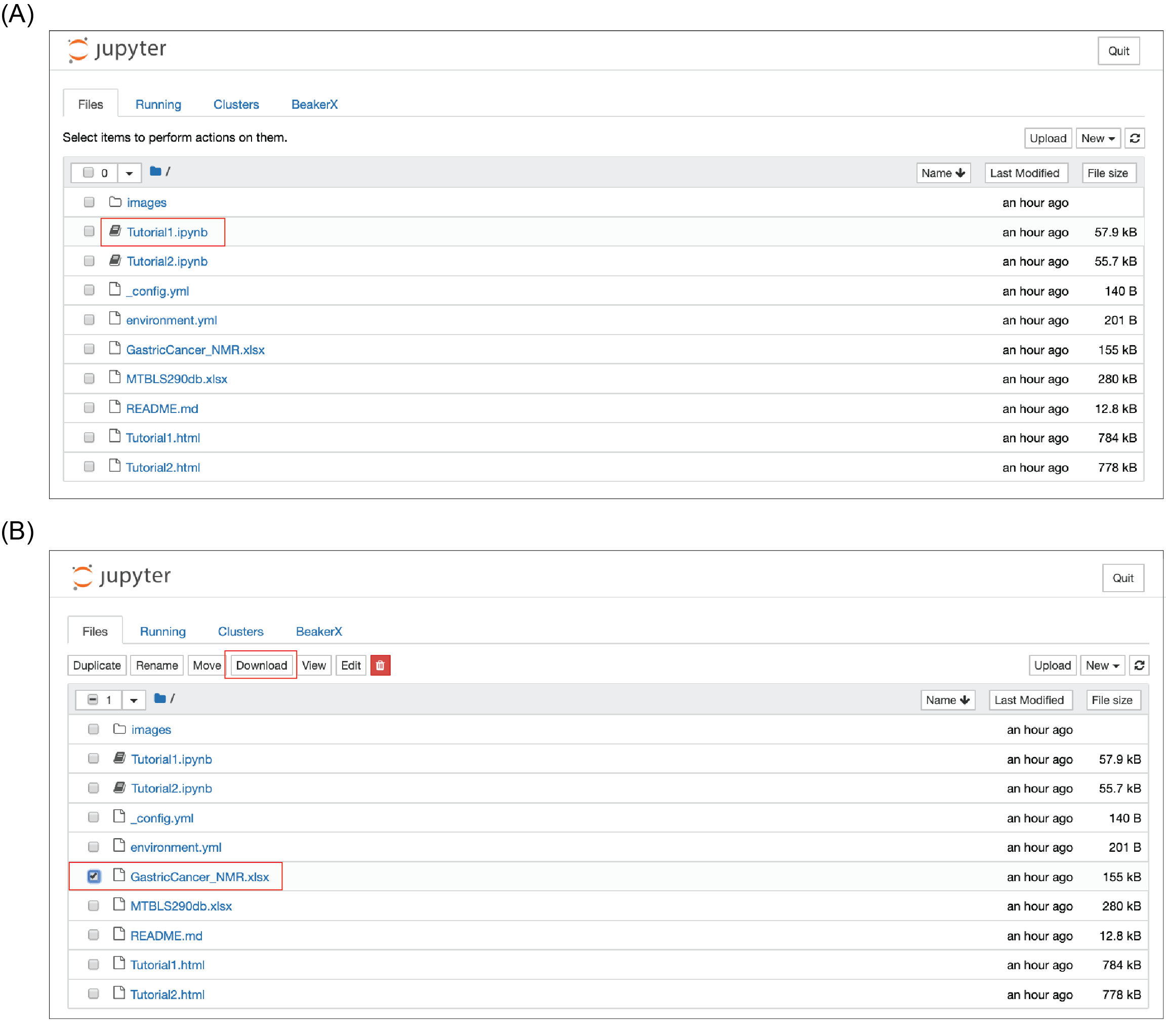


**Supplementary Figure 1. Jupyter Notebook landing page.** (A) The Jupyter Notebook landing page containing all of the files present in this copy of the GitHub repository. To launch Tutorial #1, Click on “Tutorial1.ipynb” outlined in red. (B) To download any of the files, including the Excel workbook, select them by checking the appropriate boxes on the left side and then click “Download” at the top of the screen.

**
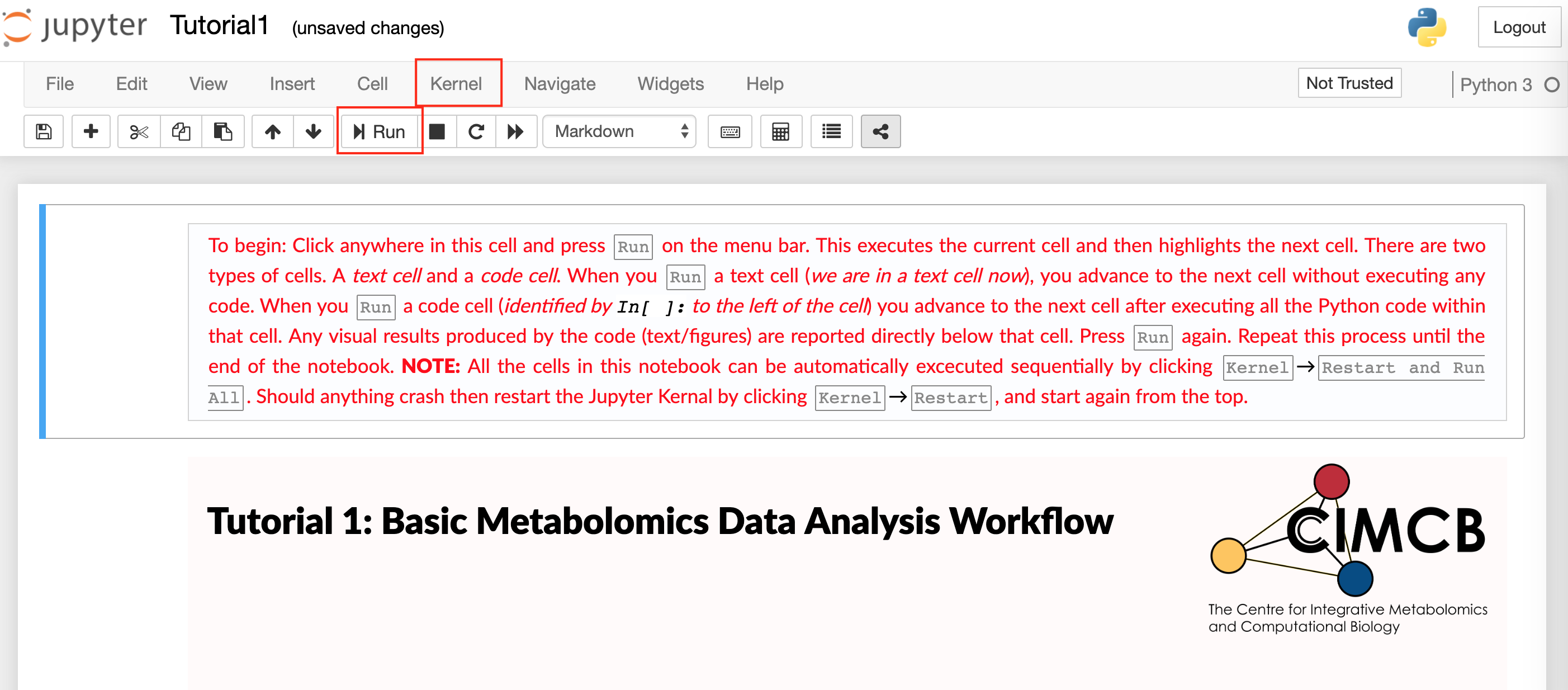
**

**Supplementary Figure 2.** The top of the Jupyter notebook Tutorial 1. At the top of the page there is a menu bar and ribbon of action buttons similar to those found in other GUI-based software, such as Microsoft Word. The interface is powerful, and it is worth taking time to become familiar with it, but for this tutorial only the “Run” button and the “Cell” and “Kernel” drop down menus are required.

(A)


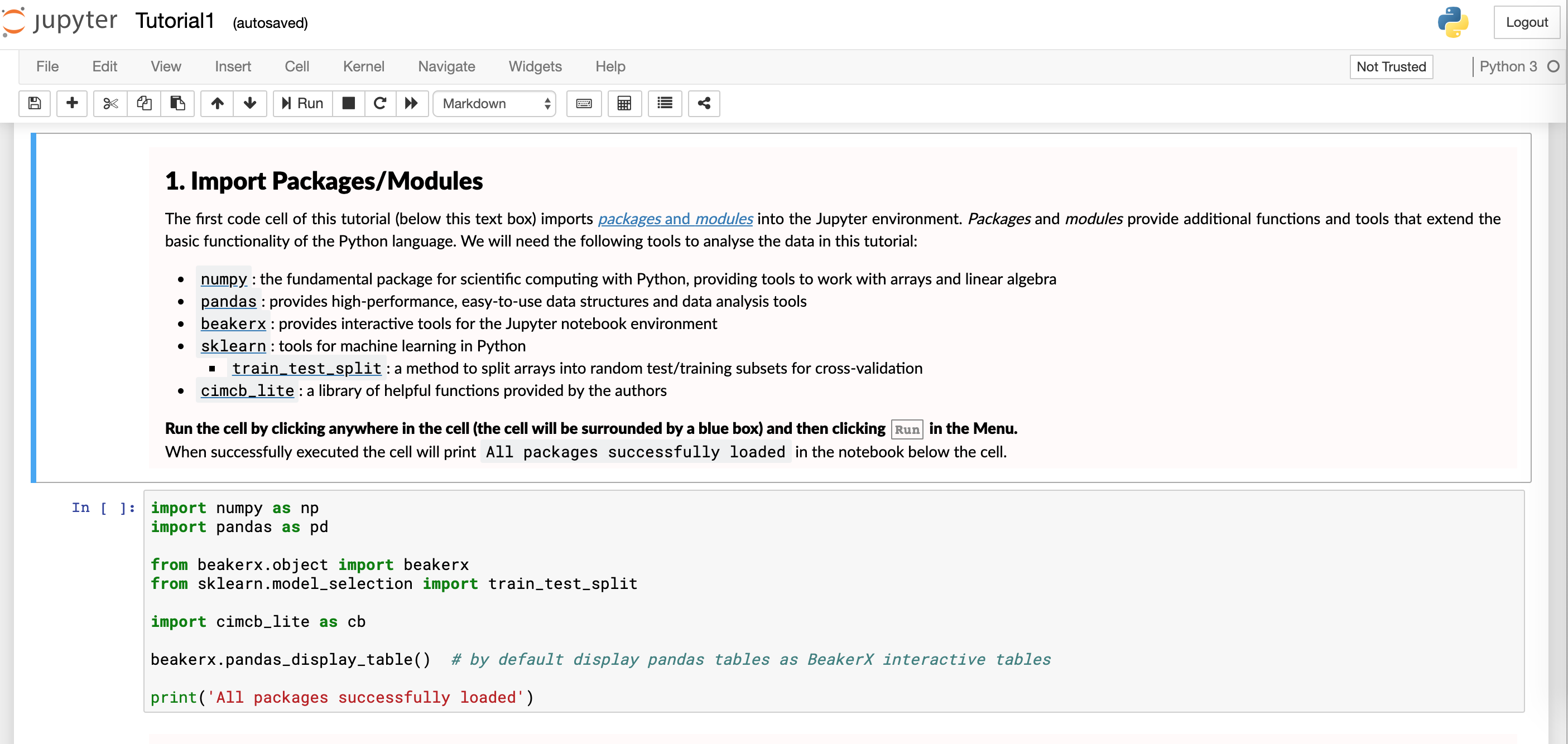


(B)


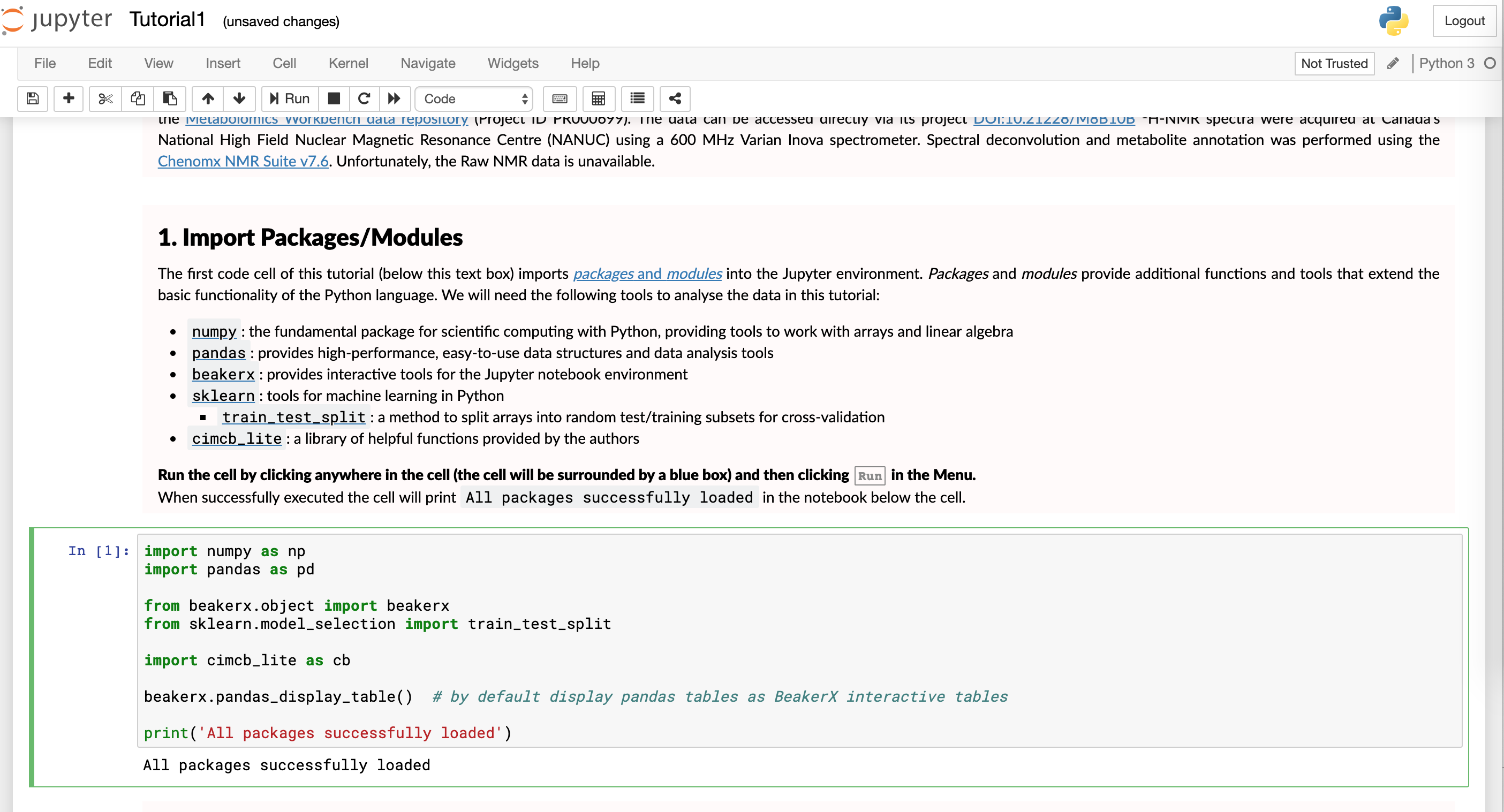


**Supplementary Figure 3.** To run a single text cell, first select it by clicking anywhere within the cell, which will then be outlined by a blue box (A). To run a single code cell, first select it by clicking anywhere within the cell, which will then be outlined by a green box (B). Once a cell is selected, the code in the cell can be executed by clicking on the “Run” button in the top menu. Multiple cells can also be run in sequence by choosing options from the dropdown list in the “Cell” menu item. The options include “Run All” (runs all the cells in the notebook, from top to bottom), and “Run all below” (run all cells below the current selection). These can be used after changing the code or values in one cell to recalculate the contents of subsequent cells in the notebook.
