# Supplementary material for: Toward collaborative open data science in metabolomics using Jupyter Notebooks and cloud computing
Source: Metabolomics. 2019 Sep 14;15(10):125. doi: 10.1007/s11306-019-1588-0 (PMC6745024; doi:10.1007/s11306-019-1588-0)
Supplement: Supplementary file 2 — Supplementary material 2 (HTML 764 kb) [file 11306_2019_1588_MOESM2_ESM.html]

Tutorial1


To begin: Click anywhere in this cell and press `Run` on the menu bar. This executes the current cell and then highlights the next cell. There are two types of cells. A *text cell* and a *code cell*. When you `Run` a text cell (*we are in a text cell now*), you advance to the next cell without executing any code. When you `Run` a code cell (*identified by In[ ]: to the left of the cell*) you advance to the next cell after executing all the Python code within that cell. Any visual results produced by the code (text/figures) are reported directly below that cell. Press `Run` again. Repeat this process until the end of the notebook. **NOTE:** All the cells in this notebook can be automatically excecuted sequentially by clicking `Kernel`→`Restart and Run All`. Should anything crash then restart the Jupyter Kernal by clicking `Kernel`→`Restart`, and start again from the top.

# Tutorial 1: Basic Metabolomics Data Analysis Workflow

This Jupyter notebook describes a typical metabolomics data analysis workflow for a study with a binary classification outcome. The main steps included are:

- Import metabolite & experimental data from an Excel sheet.
- Pooled QC-based data cleaning.
- Principal Component Analysis visualisation to check data quality.
- Two-class univariate statistics.
- Multivariate analysis using Partial Least Squares Discriminant Analysis (PLS-DA) including:
  - model optimisation (R2 vs Q2).
  - permutation testing, model prediction metrics.
  - feature importance.
  - model prediction data visualisations.
- Export statistical tables to Excel sheets.

The study used in this tutorial has been previously published as an open access article Chan et al. (2016), in the British Journal of Cancer, and the deconvolved and annotated data file deposited at the Metabolomics Workbench data repository (Project ID PR000699). The data can be accessed directly via its project DOI:10.21228/M8B10B 1H-NMR spectra were acquired at Canada’s National High Field Nuclear Magnetic Resonance Centre (NANUC) using a 600 MHz Varian Inova spectrometer. Spectral deconvolution and metabolite annotation was performed using the Chenomx NMR Suite v7.6. Unfortunately, the Raw NMR data is unavailable.

## 1. Import Packages/Modules

The first code cell of this tutorial (below this text box) imports *packages* and *modules* into the Jupyter environment. *Packages* and *modules* provide additional functions and tools that extend the basic functionality of the Python language. We will need the following tools to analyse the data in this tutorial:

- `numpy`: the fundamental package for scientific computing with Python, providing tools to work with arrays and linear algebra
- `pandas`: provides high-performance, easy-to-use data structures and data analysis tools
- `beakerx`: provides interactive tools for the Jupyter notebook environment
- `sklearn`: tools for machine learning in Python
  - `train_test_split`: a method to split arrays into random test/training subsets for cross-validation
- `cimcb_lite`: a library of helpful functions provided by the authors

**Run the cell by clicking anywhere in the cell (the cell will be surrounded by a blue box) and then clicking `Run` in the Menu.**   
When successfully executed the cell will print `All packages successfully loaded` in the notebook below the cell.

In [1]:

```
import numpy as np
import pandas as pd

from beakerx.object import beakerx
from sklearn.model_selection import train_test_split

import cimcb_lite as cb

beakerx.pandas_display_table()  # by default display pandas tables as BeakerX interactive tables

print('All packages successfully loaded')
```

```
All packages successfully loaded
```

## 2. Load Data and Peak sheet

This workflow requires data to be uploaded as a Microsoft Excel file, using the Tidy Data framework (i.e. each column is a variable, and row is an observation). As such, the Excel file should contain a *Data Sheet* and *Peak Sheet*. The *Data Sheet* contains all the metabolite concentrations and metadata associated with each observation (requiring the inclusion of the columns: *Idx*, *SampleID*, and *Class*). The *Peak Sheet* contains all the metadata pertaining to each measured metabolite (requiring the inclusion of the columns: *Idx*, *Name*, and *Label*). Please inspect the Excel file used in this tutorial before proceeding.

The code cell below loads the *Data* and *Peak* sheets from an Excel file, using the CIMCB helper function `load_dataXL()`. When this is complete, you should see confirmation that Peak (stored in the `Peak` worksheet in the Excel file) and Data (stored in the `Data` worksheet in the Excel file) tables have been loaded:

```
Loadings PeakFile: Peak
Loadings DataFile: Data
Data Table & Peak Table is suitable.
TOTAL SAMPLES: 140 TOTAL PEAKS: 149
Done!
```

Once loaded, the data is available for use in *variables* called `dataTable` and `peakTable`.

In [2]:

```
# The path to the input file (Excel spreadsheet)
filename = 'GastricCancer_NMR.xlsx'

# Load Peak and Data tables into two variables
dataTable, peakTable = cb.utils.load_dataXL(filename, DataSheet='Data', PeakSheet='Peak')
```

```
Loadings PeakFile: Peak
Loadings DataFile: Data
Data Table & Peak Table is suitable.
TOTAL SAMPLES: 140 TOTAL PEAKS: 149
Done!
```

### 2.1 Display the `Data` table

The `dataTable` table can be displayed interactively so we can inspect and check the imported values. To do this, we use the `display()` function. For this example the imported data consists of 140 samples and 149 metabolites.

Note that each row describes a single urine sample, where:

- Columns **M1** ... **M149** descibe metabolite concentrations.
- Column **SampleType** indicates whether the sample was a pooled QC or a study sample.
- Column **Class** indicates the clincal outcome observed for that individual: *GC* = Gastric Cancer , *BN* = Benign Tumor , *HE* = Healthy Control
.

In [3]:

```
display(dataTable) # View and check the dataTable
```

### 2.2 Display the `Peak` table

The `peakTable` table can also be displayed interactively so we can inspect and check the imported values. To do this, we again use the `display()` function. For this example the imported data consists of 149 metabolites (the same as in the `dataTable` data)

Each row describes a single metabolite, where

- Column **Idx** is a unique metabolite index.
- Column **Name** is the column header corresponding to this metabolite in the `dataTable` table.
- Column **Label** provides a unique name for the metabolite (or a `uNNN` identifier)
- Column **Perc\_missing** indicates what percentage of samples do not contain a measurement for this metabolite (missing data).
- Column **QC\_RSD** is a quality score representing the variation in measurements of this metabolite across all samples.

In [4]:

```
display(peakTable) # View and check PeakTable
```

## 3. Data Cleaning

It is good practice to assess the quality of your data, and remove (clean out) any poorly measured metabolites, before performing any statistical or machine learning modelling Broadhurst *et al.* 2018. For the Gastric Cancer NMR data set used in this example we have already calculated some basic statistics for each metabolite and stored them in the Peak table. In this notebook we keep only metabolites that meet the following criteria:

- a QC-RSD less than 20%
- fewer than 10% of values are missing

When the data is cleaned, the number of remaining peaks will be reported.

In [5]:

```
# Create a clean peak table 

rsd = peakTable['QC_RSD']
percMiss = peakTable['Perc_missing']
peakTableClean = peakTable[(rsd < 20) & (percMiss < 10)]

print("Number of peaks remaining: {}".format(len(peakTableClean)))
```

```
Number of peaks remaining: 52
```

## 4. PCA - Quality Assessment

To provide a multivariate assesment of the quality of the cleaned data set it is good practice to perform a simple Principal Component Analysis (PCA), after suitable transforming & scaling. The PCA score plot is typically labelled by sample type (i.e. quality control (QC) or biological sample (Sample)). Data of high quality will have QCs that cluster tightly compared to the biological samples Broadhurst *et al.* 2018.

First the metabolite data matrix is extracted from the `dataTable`, and transformed & scaled:

- A new variable `peaklist` is created, to hold the names (M1...Mn) of the metabolites to be used in subsequent statistical analysis
- The peak data for all samples, corresponding to this list, is extracted from the `dataTable` table, and placed in a matrix `X`
- The values in `X` are log-transformed (`Xlog`)
- The helper function `cb.utils.scale()` is used to scale the log-transformed data (`Xscale`)
- Missing values are imputed using a *k*-nearest neighbour approach (with three neighbours) to give the table `Xknn`

The transformed & scaled dataset `Xknn` is used as input to PCA, using the helper function `cb.plot.pca()`. This returns plots of PCA scores and PCA loadings, for interpretation and quality assessment.

In [6]:

```
# Extract and scale the metabolite data from the dataTable 

peaklist = peakTableClean['Name']                   # Set peaklist to the metabolite names in the peakTableClean
X = dataTable[peaklist].values                      # Extract X matrix from dataTable using peaklist
Xlog = np.log10(X)                                  # Log scale (base-10)
Xscale = cb.utils.scale(Xlog, method='auto')        # methods include auto, pareto, vast, and level
Xknn = cb.utils.knnimpute(Xscale, k=3)              # missing value imputation (knn - 3 nearest neighbors)

print("Xknn: {} rows & {} columns".format(*Xknn.shape))

cb.plot.pca(Xknn,
            pcx=1,                                                  # pc for x-axis
            pcy=2,                                                  # pc for y-axis
            group_label=dataTable['SampleType'])                    # labels for Hover in PCA loadings plot
```

```
Xknn: 140 rows & 52 columns
```

Loading BokehJS ...

## 5. Univariate Statistics for comparison of Gastric Cancer (`GC`) vs Healthy Controls (`HE`)

The data set uploaded into `dataTable` describes the 1H-NMR urine metabolite profiles of individuals classified into three distinct groups: `GC` (gastric cancer), `BN` (benign), and `HE` (healthy). For this workflow we are interested in comparing only the differences in profiles between individuals classified as `GC` or `HE`.

The helper function `cb.utils.univariate_2class()` will take as input a data table where the observations represent data from two groups, and a corresponding table of metabolite peak information, and produce as output summary statistics of univariate comparisons between the two groups. The output is returned as a `pandas` dataframe, describing output from statistical tests such as Student's *t*-test and Shapiro-Wilks, and summaries of data quality, like the number and percentage of missing values.

First, we reduce the data in `dataTable` to only those observations for `GC` and `HE` samples, and we define the `GC` class to be a positive outcome, in the variable `pos_outcome`. Next, we pass the reduced dataset and the cleaned `peakTable` to `cb.utils.univariate_2class()`, and store the returned dataframe in a new variable called `statsTable`. This is then displayed as before for interactive inspection and interpretation.

In [7]:

```
# Select subset of Data for statistical comparison
dataTable2 = dataTable[(dataTable.Class == "GC") | (dataTable.Class == "HE")]  # Reduce data table only to GC and HE class members
pos_outcome = "GC"

# Calculate basic statistics and create a statistics table.
statsTable = cb.utils.univariate_2class(dataTable2,
                                        peakTableClean,
                                        group='Class',                # Column used to determine the groups
                                        posclass=pos_outcome,         # Value of posclass in the group column
                                        parametric=True)              # Set parametric = True or False

# View and check StatsTable
display(statsTable)
```

It is useful to have this interactive view, but the output will disappear when we close the notebook. To store the output in a more persistent format, such as an Excel spreadsheet, we can use the *methods* that are built in to `pandas` dataframes.

To save a `pandas` dataframe to an Excel spreadsheet file as a single sheet, we use the dataframe's `.to_excel()`method, and provide the name of the file we want to write to (and optionally a name for the sheet). We do not want to keep the dataframe's own index column, so we also set `index=False`.

The code in the cell below will write the contents of `statsTable` to the file `stats.xlsx`.

In [8]:

```
# Save StatsTable to Excel
statsTable.to_excel("stats.xlsx", sheet_name='StatsTable', index=False)
print("done!")
```

```
done!
```

## 6. Machine Learning

The remainder of this tutorial will describe the use of a 2-class Partial Least Squares-Discriminant Analysis (PLS-DA) model to identify metabolites which, when combined in a linear equation, are able to classify unknown samples as either `GC` and `HE` with a measurable degree of certainty.

### 6.1 Splitting data into Training and Test sets.

Multivariate predictive models are prone to overfitting. In order to provide some level of independent evaluation it is common practice to split the source data set into two parts: **training set** and **test set**. The model is then optimised using the training data and independently evaluated using the test data. The true effectiveness of a model can only be assessed using the test data (Westerhuis *et al.* 2008, Xia *et al.* 2012). It is vitally important that both the training and test data are equally representative of the the sample population (in our example the urine metabotype of *Gastric Cancer* and the urine metabotype of *Healthy Control*). It is typical to split the data using a ratio of 2:1 (⅔ training, ⅓ test) using stratified random selection. If the purpose of model-building is exploratory, or sample numbers are small, this step is often ignored; however, care must be taken in interpreting a model that has not been tested on a dataset that is independent of the data it was trained on.

We use the `dataTable2` dataframe created above, which contains a subset of the complete data suitable for a 2-class comparision (`GC` vs `HE`). Our goal is to split this dataframe into a *training* subset (`dataTrain`) which will be used to train our model, and a *test* set (`dataTest`), which will be used to evaluate the trained model. We will split the data such that number of *test* set samples is 25% of the the total. To do this, we will use the `scikit-learn` module's `train_test_split()` function.

First, we need to ensure that the sample split - though random - is *stratified* so that the class membership is *balanced* to the same proportions in both the test and training sets. In order to do this, we need to supply a binary vector indicating stratification group membership.

The `train_test_split()` function expects a *binary* (`1`/`0`) list of *positive*/*negative* **outcome** indicators, not the `GC`/`HE` classes that we have. We convert the class information for each sample in `dataTable2` into `Y`, a list of `1`/`0` values, in the code cell below.

In [9]:

```
# Create a Binary Y vector for stratifiying the samples
outcomes = dataTable2['Class']                                  # Column that corresponds to Y class (should be 2 groups)
Y = [1 if outcome == 'GC' else 0 for outcome in outcomes]       # Change Y into binary (GC = 1, HE = 0)  
Y = np.array(Y)                                                 # convert boolean list into to a numpy array
```

Now that we have the dataset (`dataTable2`) and the list of binary outcomes (`Y`) for stratification, we can use the `train_test_split()` function in the code cell below.

Once the training and test sets have been created, summary output will be printed:

```
DataTrain = 62 samples with 32 positive cases.
DataTest = 21 samples with 11 positive cases.
```

Two new dataframes and two new lists are created:

- `dataTrain`: training data set (dataframe)
- `dataTest`: test dataset (dataframe)
- `Ytrain`: known outcomes for the training dataset
- `Ytest`: known outcomes for the test dataset

In [10]:

```
# Split dataTable2 and Y into train and test (with stratification)
dataTrain, dataTest, Ytrain, Ytest = train_test_split(dataTable2, Y, test_size=0.25, stratify=Y,random_state=10)

print("DataTrain = {} samples with {} positive cases.".format(len(Ytrain),sum(Ytrain)))
print("DataTest = {} samples with {} positive cases.".format(len(Ytest),sum(Ytest)))
```

```
DataTrain = 62 samples with 32 positive cases.
DataTest = 21 samples with 11 positive cases.
```

### 6.2. Determine optimal number of components for PLS-DA model

The most common method to determine the optimal PLS-DA model configuration without overfitting is to use k-fold cross-validation. For PLS-DA, this will be a linear search of models having *$1$ to $N$* latent variables (components).
First, each PLS-DA configuration is trained using all the available data (`XTknn` and `Ytrain`). The generalised predictive ability of that model is then evaluated using the same data - typically by calculating the coefficient of determination $R^2$. This will generate $N$ evaluation scores ($R^2\_1,R^2\_2 ... R^2\_N$).
The training data is then split into *k* equally sized subsets (folds). For each of the PLS-DA configurations, $k$ models are built, such that each model is trained using $k-1$ *folds* and the remaining 1-fold is applied to the model and model predictions are recorded. The modeling process is implemented such than that after $k$ models each fold will have been \*'held-out'\* only once.
The generalised predictive ability of the model is then evaluated by comparing the \*'held-out'\* model predictions to the expected classification (cross-validated coefficient of determination - $Q^2$). This will generate $N$ *cross-validated* evaluation scores scores ($Q^2\_1,Q^2\_2 ... Q^2\_N$). If the values for $R^2$ and $Q^2$ are plotted against model complexity (number of latent variables), typically the value of $Q^2$ will be seen to rise and then fall. The point at which the $Q^2$ value begins to diverge from the $R^2$ value is considered the point at which the optimal number of components has been met without overfitting.

In this section, we perform 5-fold cross-validation using the training set we created above (`dataTrain`) to determine the optimal number of components to use in our PLS-DA model.

First, in the cell below we extract and scale the training data in `dataTrain` the same way as we did for PCA quality assessment in section 4 (log-transformation, scaling, and k-nearest-neighbour imputation of missing values).

In [11]:

```
# Extract and scale the metabolite data from the dataTable
peaklist = peakTableClean['Name']                           # Set peaklist to the metabolite names in the peakTableClean
XT = dataTrain[peaklist]                                    # Extract X matrix from DataTrain using peaklist
XTlog = np.log(XT)                                          # Log scale (base-10)
XTscale = cb.utils.scale(XTlog, method='auto')              # methods include auto, pareto, vast, and level
XTknn = cb.utils.knnimpute(XTscale, k=3)                    # missing value imputation (knn - 3 nearest neighbors)
```

Now we use the `cb.cross_val.kfold()` helper function to carry out 5-fold cross-validation of a set of PLS-DA models configured with different numbers of latent variables (1 to 6). This helper function is generally applicable, and the values being passed here are:

- `model`: the Python class describing the statistical model to train and validate. Here, this is `cb.model.PLS_SIMPLS`, a PLS model using the SIMPLS algorithm.
- `X`: the training data set (`XTknn`)
- `Y`: the known outcomes corresponding to the dataset in `X` (`Ytrain`)
- `param_dict`: a dictionary describing key:value pairs where the key is a parameter that is passed to the model, and the value is a collection of individual values to be passed to that parameter.
- `folds`: the number of folds in the cross-validation
- `bootnum`: the number of bootstrap samples used in computing confidence intervals

The `cb.cross_val.kfold()` function returns an object that we store in the `cv` variable. To actually run the cross-validation, we call the `cv.run()` method of this object. When the cell is run, a progress bar will appear:

```
Kfold: 100%|██████████| 100/100 [00:02<00:00, 33.71it/s]
```

In [12]:

```
# initalise cross_val kfold (stratified) 
cv = cb.cross_val.kfold(model=cb.model.PLS_SIMPLS,                   # model; we are using the PLS_SIMPLS model
                        X=XTknn,
                        Y=Ytrain,
                        param_dict={'n_components': [1,2,3,4,5,6]},  # The numbers of latent variables to search                
                        folds=5,                                     # folds; for the number of splits (k-fold)
                        bootnum=100)                                 # num bootstraps for the Confidence Intervals

# run the cross validation
cv.run()
```

```
Kfold: 100%|██████████| 100/100 [00:04<00:00, 23.52it/s]
```

The object stored in the `cv` variable also has a `.plot()` method, which renders two views of $R^2$ and $Q^2$ statistics: difference ($R^2 - Q^2$), and absolute values of both metrics against the number of components, to aid in selecting the optimal number of components.

The point at which the $Q^2$ value begins to diverge from the $R^2$ value is considered to be the point at which the optimal number of components has been met without overfitting. In this case, the plots clearly indicate that the optimal number of latent variables in our model is two.

In [13]:

```
cv.plot() # plot cross validation statistics
```

Loading BokehJS ...

### 6.3 Train and evaluate PLS-DA model

Now we have determined that the optimal number of components for this example data set is 2, we create a PLS-DA model with 2 latent variables, and evaluate its predictive ability. The implementation of PLS we use is the `PLS_SIMPLS` class from the CIMCB helper module. We first create a PLS model object with two components, in the variable `modelPLS`:

In [14]:

```
modelPLS = cb.model.PLS_SIMPLS(n_components=2)  # Initalise the model with n_components = 2
```

Next we fit the model on the `XTknn` training dataset, with the values in `Ytrain` as the known response variables. We do this by calling the model's `.train()` method, with the predictor and response variables.

This returns a list of values that are the *predicted* response variables, after model fitting.

In [15]:

```
Ypred = modelPLS.train(XTknn, Ytrain)  # Train the model
```

Finally, we call the trained model's `.evaluate()` method, passing a *classification cutoff score* from which a standard set of model evaluations will be calculated from the model predictions ($R^2$, Mann-Whitney p-value, Area under ROC curve, Accuracy, Precision, Sensitivity, Specificity). The model perfomance is also visualised using the following plots:

- a violin plot showing the distributions of negative and positive responses as violin plots and box-whisker plots, with an overlay of the predicted cutoff score that discriminates between classes (dashed line).
- a probability density function plot for each response type, with overlaid predicted cutoff score (dashed line)
- ROC curve for the classifier, with 95% confidence interval (lighter shaded area), and the performance indicated with 95% CI.

From these plots and the table we find that the trained classifier performs acceptably well.

In [16]:

```
# Evaluate the model 
modelPLS.evaluate(cutoffscore=0.5)
```

Loading BokehJS ...

### 6.4. Perform a permutation test for the PLS-DA model

The reliability of our trained model can be assessed using a *permutation test*. In this test, the original data is randomised (*permuted* or 'shuffled') so that the predictor variables and response variables are mixed, and a new model is then trained and tested on the shuffled data. This is repeated many times so that the behaviour of models constructed from "random" data can be fairly assessed.

We can be confident that our model is being trained on relevant and meaningful features of the original dataset if the $R^2$ and $Q^2$ values generated from these models (with randomised data) are much lower than those found for our model trained on the original data.

The PLS model we are using from the CIMCB module has a `.permutation_test()` method that can perform this analysis for us. It returns a pair of graphs that can be used to interpret model performance.

- $R^2$ and $Q^2$ against "correlation of permuted data against original data"
- probability density functions for $R^2$ and $Q^2$, with the $R^2$ and $Q^2$ values found for the model trained on original data presented as ball-and-stick.

We see that the models trained on randomised/shuffled data have much lower $R^2$ and $Q^2$ values than the models trained on the original data, so we can be confident that the model represents meaningful features in the original dataset.

In [17]:

```
modelPLS.permutation_test(nperm=100) #nperm refers to the number of permutations
```

```
Permutation Resample: 100%|██████████| 100/100 [00:00<00:00, 102.18it/s]
```

Loading BokehJS ...

### 6.5. Plot latent variable projections for PLS-DA model

The PLS model also provides a `.plot_projections()` method, so we can visually inspect characteristics of the fitted latent variables. This returns a grid of plots:

- The diagonal shows probability density functions of each latent variable (LV) for each response class. The first latent variable (LV1) is at the top left of the plot.
- The upper triangle shows ROC curves for each optimal discriminating pairwise combination of LVx and LVy scores
- The lower triangle shows scatterplots of the scores for LVy against LVx, with a solid line indicating the direction of maximum discrimination

Where only one latent variable is fitted, a similar plot is produced to that with the `.evaluate()` method, with the addition of a scatterplot of latent variable scores)

In [18]:

```
modelPLS.plot_projections(label=dataTrain[['Idx','SampleID']], size=12) # size changes circle size
```

Loading BokehJS ...

### 6.6. Plot feature importance (Coefficient plot and VIP) for PLS-DA model

Now that we have built a model and established that it represents meaningful features of the dataset, we determine the importance of specific peaks to the model's discriminatory power.

To do this, in the cell below we use the PLS model's `plot_featureimportance()` method to render scatterplots of the PLS regression *coefficient* values for each metabolite, and *Variable Importance in Projection* (VIP) plots. The coefficient values provide information about the contribution of the peak to either a negative or positive classification for the sample, and peaks with VIP greater than unity (1) are considered to be "important" in the model.

We could generate these plots for the model as it was trained, but we would prefer to have an estimate of the robustness of these values, so we generate bootstrapped confidence intervals with the model's `.calc_bootci()` method. Any metabolite coefficient with a confidence interval crossing the zero line is considered non-significant, and thus not "important" to the model.

The `.plot_featureimportance()` method renders the two scatterplots, and also returns a new dataframe reporting these values, and their confidence intervals, which we capture in the variable `peakSheet`.

In [19]:

```
# Calculate the bootstrapped confidence intervals 
modelPLS.calc_bootci(type='bca', bootnum=200)                # decrease bootnum if it this takes too long on your machine

# Plot the feature importance plots, and return a new Peaksheet 
peakSheet = modelPLS.plot_featureimportance(peakTableClean,
                                            peaklist,
                                            ylabel='Label',  # change ylabel to 'Name' 
                                            sort=False)      # change sort to False
```

```
Bootstrap Resample: 100%|██████████| 200/200 [00:00<00:00, 1428.59it/s]
Jackknife Resample: 100%|██████████| 62/62 [00:00<00:00, 1318.42it/s]
```

Loading BokehJS ...

### 6.7. Test model with new data (using test set from section 6.1)

So far, we have trained and tested our PLS classifier on a single training dataset. This risks *overfitting* as we could be optimising the performance of the model on this dataset such that it cannot *generalise*, in the sense that it may not perform as well on a dataset that it has not already seen.

To see if the model can *generalise*, we must test our trained model using a new dataset that it has not already encountered. In section 6.1 we divided our original complete dataset into four components: `datatrain`, `Ytrain`, `dataTest` and `Ytest`. Our trained model has not seen the `dataTest` and `Ytest` values that we have *held out*, so these can be used to evaluate model preformance on new data.

We begin by transforming and scaling this *holdout* dataset in the same way as we did for the training data. To do this, we first find the mean and variance of our transformed training data set `XTlog` with the `cb.utils.scale()` function, so that we can use these values to scale the *holdout* data.

In [20]:

```
# Get mu and sigma from the training dataset to use for the Xtest scaling
mu, sigma  = cb.utils.scale(XTlog, return_mu_sigma=True)
```

Next, we extract the peak data for our holdout `dataTest` set, and put this in the variable `XV`. As before, we take the log transform (`XVlog`), scale the data in the same way as the training data (`XVscale`; note that we specify `mu` and `sigma` as calculated above), and impute missing values to give the final *holdout* test set `XVknn`.

In [21]:

```
# Pull of Xtest from DataTest using peaklist ('Name' column in PeakTable)
peaklist = peakTableClean.Name
XV = dataTest[peaklist].values

# Log transform, unit-scale and knn-impute missing values for Xtest
XVlog = np.log(XV)
XVscale  = cb.utils.scale(XVlog, method='auto', mu=mu, sigma=sigma)
XVknn = cb.utils.knnimpute(XVscale, k=3)
```

Now we predict a new set of response variables from `XVknn` as input, using our trained model and its `.test()` method, and then evaluate the performance of model prediction against the known values in `Ytest` using the `.evaluate()` method (as in section 6.3).

Three plots are generated, showing comparisons of the performance of the model on training and holdout test datasets.

- a violin plot showing the distribution of known positive and negative in both training and test sets, and the class cut-off (dotted line)
- probability density functions for positive and negative classes in the training and test sets (the training set datapoints are rendered as more opaque than the test set data in this figure)
- ROC curves of model performance on training and test sets

A table of performance metrics for both datasets is shown below the figures.

In [22]:

```
# Calculate Ypredicted score using modelPLS.test
YVpred = modelPLS.test(XVknn)

# Evaluate Ypred against Ytest
evals = [Ytest, YVpred]    # alternative formats: (Ytest, Ypred) or np.array([Ytest, Ypred])
#modelPLS.evaluate(evals, specificity=0.9)
modelPLS.evaluate(evals, cutoffscore=0.5)
```

Loading BokehJS ...

### 6.8. Export results to Excel

Finally, we will save our results in a persistent Excel spreadsheet.

Unlike section 5, we want to save two sheets in a single Excel workbook called `modelPLS.xlsx`. We want to save one sheet showing the holdout test data (with results from `YVpred`), and a separate sheet showing the peaks with their residual coefficients and VIP scores.

Firstly, we generate a dataframe containing the test dataset and the model's predictions. This will have columns for

- `idx`: sample index
- `SampleID`: sample ID
- `class`: sample class (`GC` or `HE`)
- `YPred`: predicted response variable from the trained model

In [23]:

```
# Save DataSheet as 'Idx', 'SampleID', and 'Class' from DataTest
dataSheet = dataTest[["Idx", "SampleID", "Class"]].copy()

# Add 'Ypred' to Datasheet
dataSheet['Ypred'] = YVpred

display(dataSheet) # View and check the dataTable
```

In section 5 we saved a single dataframe to an Excel workbook, as a single worksheet. Here, we want to save two worksheets. This means we can't use the `.to_excel()` method of a dataframe directly to write twice to the same file. Instead, we must create a `pd.ExcelWriter` object, and add each dataframe in turn to this object. When we are finished adding datframes, we can use the object's `.save()` method to write the Excel workbook with several worksheets (one per dataframe) to a single file.

In [24]:

```
# Create an empty excel workbook
writer = pd.ExcelWriter("modelPLS.xlsx")     # provide the filename for the Excel file

# Add each dataframe to the workbook in turn, as a separate worksheet
dataSheet.to_excel(writer, sheet_name='Datasheet', index=False)
peakSheet.to_excel(writer, sheet_name='Peaksheet', index=False)

# Write the Excel workbook to disk
writer.save()

print("Done!")
```

```
Done!
```

Congratulations! You have reached the end of tutorial 1.

In [ ]:

```

```
