# Supplementary material for: Toward collaborative open data science in metabolomics using Jupyter Notebooks and cloud computing
Source: Metabolomics. 2019 Sep 14;15(10):125. doi: 10.1007/s11306-019-1588-0 (PMC6745024; doi:10.1007/s11306-019-1588-0)
Supplement: Supplementary file 3 — Supplementary material 3 (HTML 760 kb) [file 11306_2019_1588_MOESM3_ESM.html]

Tutorial2


To begin: Click anywhere in this cell and press `Run` on the menu bar. This executes the current cell and then highlights the next cell. There are two types of cell. A *text cell* and a *code cell*. When you `Run` a text cell (*we are in a text cell now*), you advance to the next cell without executing any code. When you `Run` a code cell (*identified by In[ ]: to the left of the cell*) you advance to the next cell after executing all the Python code within that cell. Any visual results produced by the code (text/figures) are reported directly below that cell. Press `Run` again. Repeat this process until the end of the notebook. **NOTE:** All the cells in this notebook can be automatically executed sequentially by clicking `Kernel`→`Restart and Run All`. Should anything crash then restart the Jupyter Kernel by clicking `Kernel`→`Restart`, and start again from the top.

# Tutorial 2: Interactive Metabolomics Data Analysis Workflow

The functionality of this notebook is identical to Tutorial 1, but now the text cells have been expanded into a comprehensive interactive tutorial. As before, text cells provide the metabolomics context and describe the purpose of the code in the following code cell; however, this has now been simplified to avoid complete reptition of Tutorial 1. Additional coloured text boxes are now placed throughout the workflow to help novice users navigate and understand the interactive principles of a Jupyter Notebook.

**Red boxes (cog icon) provide suggestions for changing the functionality of the subsequent code cell by editing (or substituting) one or more lines of code.**

**Green boxes (mouse icon) provide suggestions for interacting with the visual results generated by a code cell. For example, the first green box in the notebook describes how to sort and colour data in the embedded data tables.**

**Blue boxes (lightbulb icon) provide further information about the theoretical reasoning behind a block of code or visualisation. This information is not essential to understand Jupyter notebooks but may be of general educational utility and interest to new metabolomics data scientists.**

## 1. Import Packages/Modules

The first code cell of this tutorial (below this text box) imports *packages* and *modules* into the Jupyter environment. *Packages* and *modules* provide additional functions and tools that extend the basic functionality of the Python language.

- All the code embedded in this example notebook is written using the Python programming language (python.org) and is based upon extensions of popular open source packages with high levels of support.
  *Note:* a tutorial on the python programming language in itself is beyond the scope of this notebook. For more information on using Python and Jupyter Notebooks please refer to the excellent:
  Python Data Science Handbook (Jake VanderPlas, 2016), which is in itself a Jupyter Notebook deployed via Binder.

In [1]:

```
import numpy as np
import pandas as pd

from beakerx.object import beakerx
from sklearn.model_selection import train_test_split

import cimcb_lite as cb

beakerx.pandas_display_table()  # by default display pandas tables as BeakerX interactive tables

print('All packages successfully loaded')
```

```
All packages successfully loaded
```

## 2. Load Data and Peak sheet

The code cell below loads the *Data* and *Peak* sheets from an Excel file, using the CIMCB helper function `load_dataXL()`. When this is complete, you should see confirmation that Peak (stored in the `Peak` worksheet in the Excel file) and Data (stored in the `Data` worksheet in the Excel file) tables have been loaded.

- There is a second datase included with this tutorial which has been converted to standardised Tidy Data format. This data has been previously published as an article Gardlo et al. (2016) in *Metabolomics*.
  Urine samples collected from newborns with perinatal asphyxia were analysed using a Dionex UltiMate 3000 RS system coupled to a triple quadrupole QTRAP 5500 tandem mass spectrometer. The deconvoluted and annotated file is deposited at the Metabolights data repository (Project ID MTBLS290).
  Please inspect the Excel file before using it in this tutorial. To change the data set to be loaded into the notebook replace `filename = 'GastricCancer_NMR.xlsx'` with `'filename = 'MTBLS290db.xlsx'`,and press `Run` on the menu bar.
  **Note: if you change the name of the file in this code cell, you will also have to make changes to Section 5 and Section 6 (as indicated in the text cell above each) for the correct models to be built. It is probably best to come back to this excercise after finishing an initial walk-through of the complete tutorial using the default data set.**

In [2]:

```
# The path to the input file (Excel spreadsheet)
filename = 'GastricCancer_NMR.xlsx'
#filename = 'MTBLS290db.xlsx'

# Load Peak and Data tables into two variables
dataTable, peakTable = cb.utils.load_dataXL(filename, DataSheet='Data', PeakSheet='Peak')
```

```
Loadings PeakFile: Peak
Loadings DataFile: Data
Data Table & Peak Table is suitable.
TOTAL SAMPLES: 140 TOTAL PEAKS: 149
Done!
```

### 2.1 Display the `Data` table

The `dataTable` table can be displayed interactively so we can inspect and check the imported values. To do this, we use the `display()` function.

- Scroll up/down & left/right using the scroll bars
- Click on any column header to sort by that column (sort alternates between ascending and decending order)
- Click on the left side of a header column for futher options
  - for column **Class** click on *'color by unique'*
  - for column **SampleType** click on *'sort ascending'* to group all the *QC* samples together.
- Click on column header **index** to sort back into the orginal order.

In [3]:

```
display(dataTable) # View and check the dataTable
```

### 2.2. Display the `Peak` sheet

The `peakTable` table can be displayed interactively in the same way.

- Click on the column header **QC\_RSD** to sort the peaks by ascending value
- Click on the left edge of the column header **QC\_RSD** and select *'heatmap'*
- Scroll up/down to see how the "quality" of the peaks increase/decrease

In [4]:

```
display(peakTable) # View and check PeakTable
```

## 3. Data Cleaning

- Replace the code: `PeakTableClean = peakTable[(rsd < 20) & (percMiss < 10]` with: `peakTableClean = peakTable[(rsd < 10) & (percMiss < 5)]`. In doing this you will see the effect of making the data cleaning criteria more stringent. This will change the number of 'clean' metabolites.

- **Note: Changing the number of clean metabolites will significantly change the outputs from all subsequent code cells.**  
   So be sure to click on `Cell`→`Run All Below` then scroll down the notebook to see how changing this setting has changed all the cell outputs.

In [5]:

```
# Create a clean peak table 

rsd = peakTable['QC_RSD']
percMiss = peakTable['Perc_missing']
peakTableClean = peakTable[(rsd < 20) & (percMiss < 10)]

print("Number of peaks remaining: {}".format(len(peakTableClean)))
```

```
Number of peaks remaining: 52
```

## 4. PCA - Quality Assesment

To provide a multivariate assesment of the quality of the cleaned data set it is good practice to perform a simple Principal Component Analysis (PCA), after suitable transforming & scaling. The PCA score plot is typically labelled by sample type (i.e. quality control (QC) or biological sample (Sample)). Data of high quality will have QCs that cluster tightly compared to the biological samples Broadhurst *et al.* 2018.

- Hover over points in the PCA Score Plot to reveal corresponding sample information ('IDX' and 'SampleType').
- Hover over points in the PCA Loading Plot to reveal corresponding metabolite information ('Name','Label', and 'QC\_RSD').
- In the menu at the top right corner of the figure click on the 'disk' icon to save the images.
- In the menu at the top right corner of the figure click on the 'magnifying glass' icon to selct a zoom area.

- Replace the code: `XScale = cb.utils.scale(Xlog, method='auto')` with: `XScale = cb.utils.scale(Xlog, method='pareto')` This will change the type of X column scaling.
- In the PCA function call `cb.plot.pca` replace the code: `pcy=2` with: `pcy=3` to change the plot from (PC1 vs. PC2) to (PC1 vs. PC3)
- Replace the code: `group_label=dataTable['SampleType']` with: `group_label=dataTable['Class']`. The PCA scores plot will now be grouped by the data in column `Class` of the `dataTable`.

- There are four type of scaling supported by the function `cimvb.utils.scale`: `'auto'`, `'range'`, `'pareto'`, `'vast'`, and `'level'`. In the context of metabolomics these are comprehensively reviewed by van den Berg **et al** 2006.

In [6]:

```
# Extract and scale the metabolite data from the dataTable 

peaklist = peakTableClean['Name']                   # Set peaklist to the metabolite names in the peakTableClean
X = dataTable[peaklist].values                      # Extract X matrix from dataTable using peaklist
Xlog = np.log10(X)                                  # Log scale (base-10)
Xscale = cb.utils.scale(Xlog, method='auto')        # methods include auto, range, pareto, vast, and level
Xknn = cb.utils.knnimpute(Xscale, k=3)              # missing value imputation (knn - 3 nearest neighbors)

print("Xknn: {} rows & {} columns".format(*Xknn.shape))

cb.plot.pca(Xknn,
            pcx=1,                                                  # pc for x-axis
            pcy=2,                                                  # pc for y-axis
            group_label=dataTable['SampleType'])                    # labels for Hover in PCA loadings plot
```

```
Xknn: 140 rows & 52 columns
```

Loading BokehJS ...

## 5. Univariate Statistics for comparison of Gastric Cancer (`GC`) vs Healthy Controls (`HE`)

The data set uploaded into `dataTable` describes the 1H-NMR urine metabolite profiles of individuals classified into three distinct groups: `GC` (gastric cancer), `BN` (benign), and `HE` (healthy). For this specific workflow we are interested in comparing only the differences in profiles between individuals classsified as `GC` and `HE`.

- Scroll up/down using the scroll bars.
- Click on the column header to sort by that column (sort alternates between ascending and decending order).
- Click on the left side of a header column for futher options, e.g.:
  - For column **TtestStat** click on **Data Bars**.
  - For column **ShapiroPvalue** click on **Format -> exponential 5** (coverts to scientific notation).

- For data set ***GastricCancer\_NMR.xlsx*** replace the code: `dataTable[(dataTable.Class == "GC") | (dataTable.Class == "HE")]` with:   
   `dataTable[(dataTable.Class == "BN") | (dataTable.Class == "HE")]` and replace `pos_outcome = "GC"` with: `pos_outcome = "BN"`. This will allow you to perform a 2-class statistical comparison between the patients with benign tumors and healthy controls.
- **OR** for data set ***MTBLS290db.xlsx*** replace the code: `dataTable[(dataTable.Class == "GC") | (dataTable.Class == "HE")]` with: `dataTable[(dataTable.Class == "Patient") | (dataTable.Class == "Control")]` and replace `pos_outcome = "GC"` with: `pos_outcome = "Patient"`. You will now perform a 2-class statistical comparison between the unhealthy patients and healthy controls.
- In the statistical function call `cb.utils.univariate_2class` replace the code: `parametric=True` with: `parametric=False` to change the statistical test to a non-parametric Wilcoxon rank-sum test.

- **Note: Changing the outcome comparison will significantly affect the output of subsequent code cells.**  
   So be sure to click on `Cell`→`Run All Below` then scroll down the notebook to see how changing this setting has changed all the cell outputs.

In [7]:

```
# Select subset of Data for statistical comparison
dataTable2 = dataTable[(dataTable.Class == "GC") | (dataTable.Class == "HE")]  # Reduce data table only to GC and HE class members
pos_outcome = "GC"

# Calculate basic statistics and create a statistics table.
statsTable = cb.utils.univariate_2class(dataTable2,
                                        peakTableClean,
                                        group='Class',                # Column used to determine the groups
                                        posclass=pos_outcome,         # Value of posclass in the group column
                                        parametric=True)              # Set parametric = True or False

# View and check StatsTable
display(statsTable)
```

- Replace the filename `"stats.xlsx"` to `"my_stats.xlsx"`
- AND/OR replace `sheet_name='StatsTable'` with `sheet_name='myStatsTable'`

In [8]:

```
# Save StatsTable to Excel
statsTable.to_excel("stats.xlsx", sheet_name='StatsTable', index=False)
print("done!")
```

```
done!
```

## 6. Machine Learning

The remainder of this tutorial will describe the use of a 2-class Partial Least Squares-Discriminant Analysis (PLS-DA) model to identify metabolites which, when combined in a linear equation, are able to classify unknown samples as either `GC` or `HE` with a measurable degree of certainty.

### 6.1 Splitting data into Training and Test sets.

- If you have changed the comparsion groups in the default data to benign tumors (BN) vs. healthy controls (HE) then replace the code: `outcome == 'GC'` with: `outcome == 'BN'`.
- For data set ***MTBLS290db.xlsx*** replace the code: `outcome == 'GC'` with: `outcome == 'Patient'`.
- Replace the code: `train_test_split(DataTable2, Y, test_size=0.25, stratify=Y)` with: `train_test_split(DataTable2, Y, test_size=0.1, stratify=Y)`. This will decrease the number of samples in the test set. How does this affect the results?

- **Note: If you change any of the code in the following Machine Learning sections you will change the performance of all the subsequent code cells.**  
   So be sure to click on `Cell`→`Run All Below` then scroll down the notebook to see how changing this setting has changed all the cell outputs.

In [9]:

```
# Create a Binary Y vector for stratifiying the samples
outcomes = dataTable2['Class']                                  # Column that corresponds to Y class (should be 2 groups)
Y = [1 if outcome == 'GC' else 0 for outcome in outcomes]       # Change Y into binary (GC = 1, HE = 0)  
Y = np.array(Y)                                                 # convert boolean list into to a numpy array

# Split DataTable2 and Y into train and test (with stratification)
dataTrain, dataTest, Ytrain, Ytest = train_test_split(dataTable2, Y, test_size=0.25, stratify=Y, random_state=10)

print("DataTrain = {} samples with {} postive cases.".format(len(Ytrain),sum(Ytrain)))
print("DataTest = {} samples with {} postive cases.".format(len(Ytest),sum(Ytest)))
```

```
DataTrain = 62 samples with 32 postive cases.
DataTest = 21 samples with 11 postive cases.
```

### 6.2. Determine optimal number of components for PLS-DA model

In this section, we will perform 5-fold cross-validation using the training set we created above (`dataTrain`) to determine the optimal number of components to use in our PLS-DA model. First, we extract and scale the training data in `dataTrain` the same way as we did for PCA quality assessment in section 4 (log-transformation, scaling, and k-nearest-neighbour imputation of missing values).

- Replace the code: `cb.utils.scale(XTlog, method='auto')` with: `cb.utils.scale(XTlog, method='pareto')` This will change the type of X column scaling.
- Replace the code: `cb.utils.scale(XTlog, method='auto')` with: `cb.utils.scale(XT, method='auto')` This change will ignore the log transformed data (`XTlog`), and scale the raw `XT` data instead (thus missing out the log tranformation step of the data preprocessing).

In [10]:

```
# Extract and scale the metabolite data from the dataTable
peaklist = peakTableClean['Name']                           # Set peaklist to the metabolite names in the peakTableClean
XT = dataTrain[peaklist]                                    # Extract X matrix from DataTrain using peaklist
XTlog = np.log(XT)                                          # Log scale (base-10)
XTscale = cb.utils.scale(XTlog, method='auto')              # methods include auto, pareto, vast, and level
XTknn = cb.utils.knnimpute(XTscale, k=3)                    # missing value imputation (knn - 3 nearest neighbors)
```

We use the `cb.cross_val.kfold()` helper function to carry out 5-fold cross-validation of a set of PLS-DA models configured with different numbers of latent variables.

- Replace the code: `param_dict={'n_components': [1,2,3,4,5,6]}` with: `param_dict={'n_components': [1,2,3,4,5,6,7,8,9,10]}`. This will increase the range of latent variables used to build PLS-DA models from a PLS-DA model with 1 latent variable to a PLS-DA model with 10 latent variables.
- Replace the code: `folds=5` with: `folds=10`. This will change the number of folds in the k-fold cross validation.
- Replace the code: `bootnum=100` with: `bootnum=500`. This will change the number of bootstrap samples used to calculate the 95% confidence interval for the $R^2$ and $Q^2$ curves. This will drastically slow down the code execution.

- For more information on the PLS SIMPLS algorithm refer to: De Jong, S., 1993. SIMPLS: an alternative approach to partial least squares regression. Chemometrics and Intelligent Laboratory Systems, 18: 251–263
- Although it is common practice to assume the optimal number of components for the PLS-DA model is chosen when $Q^2$ is at its apex (A), this is incorrect. Overtraining starts as soon as $Q^2$ significantly deviates from the $R^2$ trajectory. If the distance between $R^2$ and $Q^2$ gets large (>0.2 or the 95% CI stop overlapping) then one has to assume that the model is already overtrained. The point at which the $Q^2$ value begins to diverge from the $R^2$ value is considered point at which the optimal number of components has been met without overfitting (B). The $R^2$ vs. $(R^2 - Q^2$) plot is provided to aid decison making.

- Hover over the green data points in each of the plots to view the corresponding $R^2$ and $Q^2$ values.
- Click on a point in one of the green plots. Notice that the two plots are linked.
- Use the menu bar at the top right of the figure to save, scroll and zoom.

In [11]:

```
# initalise cross_val kfold (stratified) 
cv = cb.cross_val.kfold(model=cb.model.PLS_SIMPLS,                   # model; we are using the PLS_SIMPLS model
                        X=XTknn,
                        Y=Ytrain,
                        param_dict={'n_components': [1,2,3,4,5,6]},  # The numbers of latent variables to search                
                        folds=5,                                     # folds; for the number of splits (k-fold)
                        bootnum=100)                                 # num bootstraps for the Confidence Intervals


cv.run()  # run the cross validation
cv.plot() # plot cross validation statistics
```

```
Kfold: 100%|██████████| 100/100 [00:04<00:00, 22.06it/s]
```

Loading BokehJS ...

### 6.3 Train and evaluate PLS-DA model

Now we have determined the optimal number of components for this data set using k-fold cross validation, we create a new PLS-DA model with the requisite number of latent variables, train the model using `XTknn` and '`YT`, then evaluate its predictive ability.

- Replace the code: `n_components=2` with: `n_components=3`. This will increase the number of latent variables used in the PLS-DA model. Notice how this changes the apparent predictive ability of the model.
- Replace the code: `cutoffscore=0.5` with: `cutoffscore=0.4` This will change the decision boundary for the classifier and alter the resulting perfomance statistics.

- Hover over the green data points in each of the plots to view extra information.
- Use the menu bar at the right of the figures to save, scroll and zoom.

In [12]:

```
modelPLS = cb.model.PLS_SIMPLS(n_components=2)  # Initalise the model with n_components = 2

Ypred = modelPLS.train(XTknn, Ytrain)  # Train the model 

modelPLS.evaluate(cutoffscore=0.5)  # Evaluate the model

modelPLS.permutation_test(nperm=100)  #nperm denotes to the number of permutations
```

Loading BokehJS ...

```
Permutation Resample: 100%|██████████| 100/100 [00:00<00:00, 102.14it/s]
```

Loading BokehJS ...

### 6.4. Plot latent variable projections for PLS-DA model

The PLS model also provides a `.plot_projections()` method, so we can visually inspect characteristics of the fitted latent variables. This returns a grid of plots:

- These plots are useful to visualise to what degree each model component (latent variable) contribute to the model's discriminative ability. In the Gastric cancer example each individual component does not perform well in isolation. It is only when combined that a good prdicitve ability is revealed. In the bottom left figure the prjection scores plot includes a solid diagonal line describing the direction of prediction and a dashed line describing the orthogonal variance. In the method orthogonal partial least squares (O-PLS) this rotation is performed automatically to aid interpretation. However, these changes only improve the interpretability, not the predictivity, of the PLS models (see Fiehnlab for further discussion)

In [13]:

```
modelPLS.plot_projections(label=
                          dataTrain[['Idx','SampleID']], size=12) # size changes circle size
```

Loading BokehJS ...

### 6.5. Plot feature importance (Coefficient plot and VIP) for PLS-DA model

Now that we have built a model and established that it represents meaningful features of the dataset, we determine the importance of specific peaks to the model's discriminatory power. To do this, in the cell below we use the PLS model's `plot_featureimportance()` method to render scatterplots of the PLS regression *coefficient* values for each metabolite, and *Variable Importance in Projection* (VIP) plots. The coefficient values provide information about the contribution of the peak to either a negative or positive classification for the sample, and peaks with VIP greater than unity (1) are considered to be "important" in the model.

- In statistics, the bootstrap procedure involves choosing random samples with replacement from a data set and calculating some statistic on those samples. The range of sample estimates you obtain enables you to establish the uncertainty of the quantity you are estimating. Sampling with replacement means that each observation in a sample is selected (and recorded) at random from the original dataset and then replaced, so it is possilbe for an observation can be selected multiple times. If the orginal data set contains N observations then each bootstrap sample contains N randomly selected observations. It has been shown that approximately 2/3 of the orginal data are include in each bootstrap sample (with 1/3 of the original data being included twice). Here we use bootstrap resampling to calculate confidence intervals for the coefficients in the PLS-DA model using the 'bootstrapping of observations' method.

- Replace the code: `type='bca'` with either `type='perc'` or `type='bc'` to change from Bias corrected and accelerated percentile method to either *Bias corrected percentile method* or *percentile method*
- Replace the code: `sort=False` with: `sort=True`. This will sort the metabolites in decending order of importance.

In [14]:

```
# Calculate the bootstrapped confidence intervals 
modelPLS.calc_bootci(type='bca', bootnum=200)                # decrease bootnum if it this takes too long on your machine

# Plot the feature importance plots, and return a new Peaksheet 
peakSheet = modelPLS.plot_featureimportance(peakTableClean,
                                            peaklist,
                                            ylabel='Label',  # change ylabel to 'Name' 
                                            sort=False)      # change sort to False
```

```
Bootstrap Resample: 100%|██████████| 200/200 [00:00<00:00, 1197.93it/s]
Jackknife Resample: 100%|██████████| 62/62 [00:00<00:00, 1052.18it/s]
```

Loading BokehJS ...

### 6.6. Test model with new data (using test set from section 6.1)

So far, we have trained and tested our PLS classifier on a single training dataset. This risks *overfitting* as we could be optimising the performance of the model on this dataset such that it cannot *generalise*, in the sense that it may not perform as well on a dataset that it has not already seen. To see if the model can *generalise*, we must test our trained model using a new dataset that it has not already encountered. In section 6.1 we divided our original complete dataset into four components: `datatrain`, `Ytrain`, `dataTest` and `Ytest`. Our trained model has not seen the `dataTest` and `Ytest` values that we have *held out*, so these can be used to evaluate model preformance on new data.

- Note: It is important that the test data is tranformed and scaled using the same parameters as the training data. If the training data is log transformed then the test data must also be log transformed, otherwise the test predictions will be inappropriate, and likely highly imprecise. Equally the scaling must be performed using the scaling factors derived from the training data (e.g. mean-centred to the traning data mean, and normalised to the training data standard deviation.

In [15]:

```
# Get mu and sigma from the training dataset to use for the Xtest scaling
mu, sigma  = cb.utils.scale(XTlog, return_mu_sigma=True)

# Pull of Xtest from DataTest using peaklist ('Name' column in PeakTable)
peaklist = peakTableClean.Name
XV = dataTest[peaklist].values

# Log transform, unit-scale and knn-impute missing values for Xtest
XVlog = np.log(XV)
XVscale  = cb.utils.scale(XVlog, method='auto', mu=mu, sigma=sigma)
XVknn = cb.utils.knnimpute(XVscale, k=3)
```

Now we predict a new set of response variables from `XVknn` as input, using our trained model and its `.test()` method, and then evaluate the performance of the prediction against the known values in `Ytest` using the `.evaluate()` method (as in section 6.3).

- Note: Although the calulcated bootstrap confidence intervals for prediciton will give an estimate of uncertainty of prediction the only way to definitively evaluate any model is with an independent test set, as shown in this plot.

In [16]:

```
# Calculate Ypredicted score using modelPLS.test
YVpred = modelPLS.test(XVknn)

# Evaluate Ypred against Ytest
evals = [Ytest, YVpred]    # alternative formats: (Ytest, Ypred) or np.array([Ytest, Ypred])
#modelPLS.evaluate(evals, specificity=0.9)
modelPLS.evaluate(evals, cutoffscore=0.5)
```

Loading BokehJS ...

### 6.7. Export results to Excel

Finally, we copy our model predictions into a table and save in a persistent Excel spreadsheet.

- Replace the filename `"modelPLS.xlsx"` to `"myModelPLS.xlsx"`
- AND/OR change `sheet_name='Datasheet'` / `sheet_name='PeakSheet'` as appropriate

In [17]:

```
# Save DataSheet as 'Idx', 'SampleID', and 'Class' from DataTest
dataSheet = dataTest[["Idx", "SampleID", "Class"]].copy()

# Add 'Ypred' to Datasheet
dataSheet['Ypred'] = YVpred

# Create an empty excel workbook
writer = pd.ExcelWriter("modelPLS.xlsx")     # provide the filename for the Excel file

# Add each dataframe to the workbook in turn, as a separate worksheet
dataSheet.to_excel(writer, sheet_name='Datasheet', index=False)
peakSheet.to_excel(writer, sheet_name='Peaksheet', index=False)

# Write the Excel workbook to disk
writer.save()

print("Done!")
```

```
Done!
```

Congratulations! You have completed tutorial 2.

In [ ]:

```

```
