# Supplementary material for: Toward collaborative open data science in metabolomics using Jupyter Notebooks and cloud computing
Source: Metabolomics. 2019 Sep 14;15(10):125. doi: 10.1007/s11306-019-1588-0 (PMC6745024; doi:10.1007/s11306-019-1588-0)
Supplement: Supplementary file 4 — Supplementary material 4 (HTML 647 kb) [file 11306_2019_1588_MOESM4_ESM.html]

Tutorial4\_Python


# Tutorial 4¶

## 1. Import Data¶

1. Import the `pandas` python module and name it `pd`
2. Load the Excel sheet "Data" from Excel file "data.xlsx" into a dataframe called `data`.
3. Display the number of rows and columns in `data`.
4. Display the first 10 rows at the top (head) of the `data` table.
5. Load the Excel sheet "Peak" from Excel file "data.xlsx" into a dataframe called `peak`.
6. Display the number of rows and columns in `peak`.
7. Display the first 10 rows at the top (head) of the `peak` table.

In [6]:

```
import pandas as pd

data = pd.read_excel('data.xlsx', sheet_name='Data') 
print("Data Table: {} rows & {} columns".format(*data.shape))
display(data.head(10)) # View data table (top 10 rows)

peak = pd.read_excel('data.xlsx', sheet_name='Peak') 
print("Peak Table: {} rows & {} columns".format(*peak.shape))
display(peak.head(10))
```

```
Data Table: 91 rows & 3087 columns
```

|  | Order | SampleType | QC | M1 | M2 | M3 | M4 | M5 | M6 | M7 | ... | M3075 | M3076 | M3077 | M3078 | M3079 | M3080 | M3081 | M3082 | M3083 | M3084 |
| --- | --- | --- | --- | --- | --- | --- | --- | --- | --- | --- | --- | --- | --- | --- | --- | --- | --- | --- | --- | --- | --- |
| 0 | 1 | QC | 1 | 1.837062e+08 | 1.296528e+08 | 3.961182e+07 | 3.732578e+07 | 5.339865e+06 | 1.031822e+08 | 9.409682e+06 | ... | 35153.206002 | 15735.886504 | 24928.165047 | 30134.444665 | 3035.247672 | 18271.101975 | 20981.783849 | 3523.418763 | 23752.440055 | 17161.698785 |
| 1 | 2 | QC | 1 | 2.030571e+08 | 1.178121e+08 | 6.361418e+07 | 6.682747e+07 | 5.572493e+06 | 9.366510e+07 | 9.941482e+06 | ... | 36701.059662 | 15590.522963 | 24776.756320 | 30250.013996 | 3052.288747 | 22116.296519 | 24543.382677 | 3389.677457 | 24262.317947 | 18949.290754 |
| 2 | 3 | Sample | 0 | 1.285728e+08 | 2.726444e+08 | 4.611692e+07 | 4.717621e+07 | 3.178726e+06 | 8.077425e+07 | 6.924520e+06 | ... | 43120.310719 | 16378.981747 | 28627.451582 | 31297.776297 | 1738.358685 | 17695.679626 | 16703.470005 | 1113.489872 | 29717.457693 | 14683.792853 |
| 3 | 4 | Sample | 0 | 1.491128e+08 | 1.955086e+08 | 5.212802e+07 | 5.114760e+07 | 3.428193e+06 | 8.211311e+07 | 7.416907e+06 | ... | 44663.545552 | 17429.459487 | 25703.703594 | 26399.809078 | 846.020446 | 15306.546820 | 15198.095655 | 1137.752119 | 26610.868749 | 11913.223789 |
| 4 | 5 | Sample | 0 | 1.590268e+08 | 3.780061e+08 | 4.238071e+07 | 4.282051e+07 | 3.186309e+06 | 1.000761e+08 | 7.030797e+06 | ... | 41770.351723 | 18467.519040 | 27371.393974 | 28374.785933 | 1959.397898 | 14904.243032 | 15625.089253 | 1101.738536 | 26496.665356 | 12750.259715 |
| 5 | 6 | Sample | 0 | 1.875314e+08 | 7.153474e+07 | 5.007826e+07 | 5.036435e+07 | 4.849348e+06 | 9.998757e+07 | 1.039036e+07 | ... | 41616.726360 | 11426.528395 | 23313.373795 | 28582.727888 | 872.115128 | 15301.063816 | 14321.556052 | 1030.238432 | 32587.650554 | 18655.811727 |
| 6 | 7 | QC | 1 | 1.983585e+08 | 1.204062e+08 | 5.190127e+07 | 5.180519e+07 | 5.348706e+06 | 8.966852e+07 | 9.605938e+06 | ... | 32839.675140 | 16262.163327 | 21298.643088 | 28065.010551 | 3277.276918 | 20071.616958 | 18198.098904 | 3047.831055 | 24997.154651 | 14494.628663 |
| 7 | 8 | Sample | 0 | 1.460268e+08 | 2.274148e+08 | 5.872350e+07 | 5.581725e+07 | 4.342786e+06 | 1.059740e+08 | 1.401639e+07 | ... | 35339.968664 | 25426.050392 | 51819.646796 | 21762.932664 | 1822.815140 | 23457.954245 | 23651.858710 | 1210.741328 | 22834.426860 | 15771.899526 |
| 8 | 9 | Sample | 0 | 1.381372e+08 | 1.269111e+08 | 5.276344e+07 | 4.936756e+07 | 3.005090e+06 | 8.101362e+07 | 1.484466e+07 | ... | 33766.256647 | 15963.341703 | 50651.507533 | 18503.876558 | 843.925809 | 16572.962662 | 16625.898352 | 1186.166100 | 25623.108950 | 13811.758227 |
| 9 | 10 | Sample | 0 | 1.601298e+08 | 4.347327e+08 | 4.883041e+07 | 4.518047e+07 | 3.326398e+06 | 7.714394e+07 | 1.239396e+07 | ... | 31442.161072 | 22600.725041 | 51901.700174 | 16804.403434 | 1522.101762 | 19948.743747 | 22690.058851 | 1229.664436 | 22637.770945 | 15009.816887 |

10 rows × 3087 columns

```
Peak Table: 3084 rows & 6 columns
```

|  | Idx | Name | Mol\_Weight | RT\_minutes | RSD | D\_Ratio |
| --- | --- | --- | --- | --- | --- | --- |
| 0 | 1 | M1 | 113.05902 | 1.276 | 5.012937 | 0.28 |
| 1 | 2 | M2 | 203.11564 | 0.950 | 9.557013 | 0.19 |
| 2 | 3 | M3 | 161.10514 | 1.428 | 16.085023 | 0.26 |
| 3 | 4 | M4 | 129.07893 | 1.400 | 18.035797 | 0.49 |
| 4 | 5 | M5 | 161.10510 | 1.272 | 19.515359 | 0.40 |
| 5 | 6 | M6 | 225.94404 | 5.923 | 6.510319 | 0.33 |
| 6 | 7 | M7 | 194.08026 | 1.169 | 2.890900 | 0.03 |
| 7 | 8 | M8 | 115.06345 | 7.067 | 4.733385 | 0.16 |
| 8 | 9 | M9 | 131.09461 | 6.220 | 6.138996 | 0.33 |
| 9 | 10 | M10 | 117.07900 | 6.971 | 4.783892 | 0.32 |

## 2. Histogram of RSD¶

1. Import the `matplotlib.pyplot` visualisation package and name it `plt`
2. Invoke the special "Jupyter Magic" command `%matplotlib inline` to help simplify plotting
3. Create a histogram of the column `peak.RSD`
4. Add an x-axis label "RSD" to the figure
5. Generate the figure using the function `plt.show()`

In [2]:

```
import matplotlib.pyplot as plt
%matplotlib inline 

plt.hist(peak.RSD, 50, density=True, facecolor='g', alpha=0.5) 
plt.xlabel('RSD', fontsize=15)
plt.show()
```

## 3. Jointplot of RSD vs. D-Ratio¶

1. Import the `seaborn` visualisation package and name it `sns`
2. Create a plot of `peak` columns `peak.RSD` vs. `peak.D-Ratio` with bivariate and distribution graphs. (automatically displayed)

In [3]:

```
import seaborn as sns

sns.jointplot(x=peak.RSD, y=peak.D_Ratio, kind='kde', color="skyblue")
```

Out[3]:

```
<seaborn.axisgrid.JointGrid at 0x10e402048>
```

## 4. PCA score plot of QC vs. Sample¶

1. Import the packages required to calculate the principal component analysis (PCA)
   - `numpy` (import as `np`)
   - `scikit-learn` packages `sklearn.decomposition.PCA`, `sklearn.preprocessing.StandardScaler`
2. Import the matplotlib package required to map colours to datapoints, to plot the PCA scores
   - `matplotlib.colors.ListedColormap`
3. Scale the data and calculate the PCA.
4. Plot PCA scores (pc1 vs pc2) labeling/coloring samples by 'QC' or 'Sample'

In [4]:

```
# Import
import numpy as np
from sklearn.decomposition import PCA
from sklearn.preprocessing import StandardScaler
from matplotlib.colors import ListedColormap

# Extract X matrix
names = peak['Name']
x = data[names].values
x = np.log(x)
x = StandardScaler().fit_transform(x)

# Create and fit PCA
pca = PCA(n_components=2)
scores = pca.fit_transform(x)
label = data['SampleType']

# Split scores into sample and QC
Sample_scores = scores[label == 'Sample',:]
QC_scores = scores[label == 'QC',:]

# Plot Sample score and QC score
fig = plt.figure(figsize=(8,8))
h1 = plt.scatter(Sample_scores[:,0],Sample_scores[:,1],edgecolors='Black', facecolors='Green',s=100,alpha=0.5)
h2 = plt.scatter(QC_scores[:,0],QC_scores[:,1], edgecolors='Black', facecolors='Red',s=100,alpha=0.5)

# Add legend, labels, and title
plt.legend((h1,h2),('Sample','QC'),fontsize=15)
plt.xlabel('PC1', fontsize=15)
plt.ylabel('PC2', fontsize=15)
plt.title('Quality Control PCA plot',fontsize=20)

# Show plot
plt.show()
```

## 5. Bubble plot of Molecular Weights vs. Retention Time (sized by RSD)¶

1. Create a scatterplot of two `peak` columns: `peak.Mol_Weight` vs. `peak.RT_minutes` (each dot is a metabolite peak).
   - make the size of the dots proportional to the RSD of the datapoint with the argument `s=peak.RSD**2/2`
   - make the dots (bubbles) transparent with the argument `alpha=0.2`
2. Add x-axis label "Molecular Weight", and y-axis label "RT minutes"
3. Add a plot title
4. Render the plot

In [5]:

```
# Scatterplot of Mol_Weight vs. RT_minute with size RSD^2/2, and colour red
fig = plt.figure(figsize=(20,16))
plt.scatter(peak.Mol_Weight, peak.RT_minutes, s=peak.RSD**2/2, alpha=0.2, edgecolors='black', c='red') 
plt.xlabel('Molecular Weight', fontsize=15)
plt.ylabel('RT minutes', fontsize=15)
plt.title('Metabolites Detected (sized by RSD)',fontsize=20)
plt.show()
```

In [ ]:

```

```

In [ ]:

```

```
